# An Opposition-Based Evolutionary Algorithm for Many-Objective Optimization with Adaptive Clustering Mechanism

**DOI:** 10.1155/2019/5126239

**Published:** 2019-05-02

**Authors:** Wan Liang Wang, Weikun Li, Yu Le Wang

**Affiliations:** Zhejiang University of Technology, Hangzhou, Zhejiang 310023, China

## Abstract

Balancing convergence and diversity has become a key point especially in many-objective optimization where the large numbers of objectives pose many challenges to the evolutionary algorithms. In this paper, an opposition-based evolutionary algorithm with the adaptive clustering mechanism is proposed for solving the complex optimization problem. In particular, opposition-based learning is integrated in the proposed algorithm to initialize the solution, and the nondominated sorting scheme with a new adaptive clustering mechanism is adopted in the environmental selection phase to ensure both convergence and diversity. The proposed method is compared with other nine evolutionary algorithms on a number of test problems with up to fifteen objectives, which verify the best performance of the proposed algorithm. Also, the algorithm is applied to a variety of multiobjective engineering optimization problems. The experimental results have shown the competitiveness and effectiveness of our proposed algorithm in solving challenging real-world problems.

## 1. Introduction

Over the last two decades, evolutionary algorithm (EA) has been proven to be prevalent and efficient to solve real-world optimization problem [1]. Some of these well-known methodologies include genetic algorithms (GAs) [2], evolution strategies (ES) [3], and ant colony optimization (ACO) [4]. However, real-world optimization problems always involve multiple objectives, which means there is no single solution when considering multiple objectives as the goal of the optimization process [5]. In this case, the solutions for a multiobjective problem(MOP), which is the main focus of the algorithm, represent the trade-offs between the objectives due to the nature of such problems [6].

For the evolutionary approach to address multiobjective optimization, which is called multiobjective evolutionary algorithm (MOEA), different varieties of the algorithms have been proposed in recent years. Among them, the algorithms inspired by nature have drawn the attention like improving strength-Pareto evolutionary algorithm (SPEA2) [7], nondominated sorting genetic algorithm version 2 (NSGA-II) [8], multiobjective particle swarm optimization (MOPSO) [9], multiobjective moth-flame algorithm [10], and multiobjective ant lion optimizer [11].

There is no doubt that MOEA has been proven to be prevalent and efficient to solve optimization problem with less than three objectives [1]. However, with the gradually rising scale of data and the pluralism of target requirements, many real-world optimization problems containing more than three objectives named “many-objective problems” (MaOPs) [12, 13] are widely appearing in engineering [14], traffic [15], and water [16]. Unfortunately, the effectiveness of previous MOEAs tends to deteriorate dramatically with the increase in the number of objectives, which has been verified in [17, 18]. This can be attributed to the situation that almost all solutions in a population become nondominated with one another, and the conflict between convergence and diversity becomes aggravated with the increasing number of objectives in MaOPs [19, 20]. Moreover, computational complexity of calculating some performance metrics and the representation and visualization of the trade-off surface are also the difficulties of MaOPs. To overcome these drawbacks, a series of many-objective evolutionary algorithms (MaOEAs) have been proposed to address these optimization problems with more than three objectives. In summary, the proposed algorithms can be roughly classified into the following four types.

### 1.1. New Domination Relation-Based Approach

As the selection criterion based on the standard dominance relationship fails to distinguish solutions in MaOPs, a number of new domination principles have been proposed to adaptively discretize the Pareto-optimal front, for instance, the *ϵ*-dominance [21, 22], CDAS-dominance [23, 24], *α*-dominance [25], fuzzy Pareto dominance [26], *L*-dominance [27], cone-domination [28, 29], and grid-based evolutionary algorithm (GrEA) [30]. Furthermore, the recently proposed generalization of Pareto optimality (GPO) [31] expands the dominance area of solutions to enhance the scalability of existing Pareto-based algorithms [32] and employs a shift-based density estimation strategy (SDE) into the dominance-based criterion, and *θ*-dominance [33, 34]introduces a new dominance relation to rank solutions, which all have been proven to be more effective than the original Pareto dominance relation. In a word, these algorithms are proposed with several variants of the Pareto dominance to enhance the selection pressure toward the PF. However, the drive toward a more aggressive selection pressure could make diversity maintenance more difficult in these new dominance-based MaOEAs.

### 1.2. Indicator-Based Approach

Aiming to obtain a desired ordering among the representative PF approximations, indicator-based MOEAs have been widely studied, for example, the indicator-based evolutionary algorithm (IBEA) [35], SMS-EMOA [36], the fast hypervolume-based evolutionary algorithm (HypE) [37], DNMOEA/HI [38], R2 indicator based [39], Δ_*p*_ [40], stochastic ranking algorithm based on multiple indicators (SRA) [41], and IGD indicator-based evolutionary algorithm (MaOEA/IGD) [42]. However, the computational cost of some performance calculation could be prohibitively expensive as the number of objectives increases.

### 1.3. Decomposition-Based Approach

These algorithms always decompose a MaOP into several single-objective optimization problems and use aggregation functions to differentiate many-objective solutions. Among the methods that use a set of weight vectors to generate multiple aggregation functions, multiobjective evolutionary algorithm based on decomposition (MOEA/D) [43] is the most representative algorithm, which aggregates the objectives of an MOP into an aggregation function with a unique weight vector. Around the MOEA/D, several variants have been proposed to strike a better balance between convergence and diversity such as I-DBEA [44], MOEA/D-DD [45], MOEA/D-DU [46], and MOEA/D-LWS [47]. However, the situation that the number of scalarizing functions is usually very limited might cause difficulties for diversity maintenance of the solution especially when compared with the exponentially increasing objective space.

### 1.4. Reference Set-Based Approach

The algorithms of this category use a set of reference solutions to measure the quality of solutions. Thus, the search process is guided by the solutions in the reference solution set. Praditwong and Yao [48] and Wang [49] proposed a novel two archive algorithm (TAA) and its improved version (Two_Arch2), in which the convergence archive (CA) can be seen as an online-updated real reference set. The recently proposed vector angle-based evolutionary algorithm (VaEA) [50] uses the population as the reference set to dynamically guide the evolutionary process. As for the algorithm using virtual reference set, NSGA-III [51, 52]is predominant which employs a set of predefined reference points to manage the diversity of the candidate solutions. Hereafter, a number of algorithms have been proposed with the reference point or vector such as the reference vector-guided evolutionary algorithm for many-objective optimization (RVEA) [53], reference vector-guided evolutionary Pareto evolutionary algorithm with reference direction (SPEAR) [54], and many-objective evolutionary optimization based on reference points (RPEA) [55].

The algorithm from the third category and fourth category becomes particularly prevalent for many-objective optimization, which could be attributed to the low cost to achieve a representative subset of the entire PF especially in a limited population size. However, most algorithms belong to these categories simply use the angle or distance solely to measure the quality of the population members with the reference set, which may lose some good solutions due to their simplex selection mechanism. Furthermore, it has been logically proved by the No Free-Lunch (NFL) theorem [56] that none of these algorithms is able to solve all optimization problems, which allows the researchers to propose new methods or improve the current algorithms for better solving the problems [33, 57]. Therefore, this paper proposes an opposition-based multiobjective evolutionary algorithm with an adaptive clustering mechanism, in short named OBEA to strengthen the selection mechanism through comprehensive consideration of the angle and the distances. The main properties of OBEA can be summarized as follows:

- A new initialization approach is designed with the assistance of the opposition-based learning (OBL) to generate the population. Due to the fact that random initialization lowers the chance of sampling better regions in algorithms, here we use the OBL to initialize the populations in stand of the previous random method. Moreover, opposition-based learning is also adapted in the evolutionary process with the aim of enhancing the probability of obtaining the better solutions.
- An adaptive clustering strategy is integrated in this algorithm. In this proposed strategy, the acute angle and perpendicular distance between the candidate solutions and the reference vectors are combined through an adaptive mechanism to cluster the candidate. Moreover, a novel selective approach is designed to dynamically select the individual with comprehensive consideration of the balance convergence and diversity.

Furthermore, an extensive comparison between the proposed OBEA with nine algorithms is implemented on 60 instances of 14 test problems taken from two well-known test suites. The results indicate that OBEA is a very promising alternative for many-objective optimization. The rest of this paper is organized as follows.  Section 2 introduces the background knowledge, and details of the proposed OBEA are described in Section 3.  Section 4 presents the numerical results of OBEA on benchmark and the detailed analysis of the proposed algorithm together with nine MaOEAs. Finally, conclusions and future work are given in Section 5.

## 2. Background

In this section, the main components of multiobjective optimization problem (MOP) are given first, which involve the basic knowledge of optimization and Pareto dominate. Next, a brief description of the reference vector is given, which is used as the underlying mechanism for solving many-objective optimization problems.

### 2.1. Multiobjective Problem

Generally, a multiobjective optimization problem can be stated as follows:

(1) $$\begin{array}{c} \begin{array}{cc} \text{minimize } & F\left(x\right)={\left(f_{1}\left(x\right),f_{2}\left(x\right),\ldots ,f_{m}\left(x\right)\right)}^{T}\\ \text{subject to} & x\in \Omega \;\subseteq \;{\mathbb{R}}^{n}, \end{array} \end{array}$$

where *x*={*x*_1_, *x*_2_,…, *x*_*n*_} is the decision vector that satisfies *x* ∈ *Ω* and *Ω* stands for the decision space. The objective function vector *F* : *Ω*⟶*ℝ*^*m*^ consists of *m* (*m* ≥ 2) objectives and *ℝ*^*m*^ refers to the objective space.

**Definition 1 .**

Given two vector *x*, *y* ∈ *Ω*, **x** is *Pareto dominate ***y** (denoted as **x** ≺ **y**) if and only if *f*_*i*_(**x**) ≤ *f*_*i*_(**y**) for each *i* ∈ (1,2,…, *m*), and ∃*j* ∈ (1,2,…, *m*), *f*_*j*_(**x**) < *f*_*j*_(**y**).

**Definition 2 .**

A decision vector $\tilde{x}\in \Omega$ is said to be *Pareto optimal*, if and only if there is no $x\in \Omega ,\;x\;≺\;\tilde{x}$.

**Definition 3 .**

The set of Pareto-optimal solutions (PS) is defined as *PS*={**x** ∈ *Ω*|*x* *is* *Pareto* *optimal*}.

**Definition 4 .**

The Pareto-optimal front (PF) is defined as *PF*={*F*(**x**)|**x** ∈ *PS*}.

### 2.2. Reference Vector

As an underlying mechanism throughout the algorithm, OBEA uses Das and Dennis's systematic approach [58], and adaptation of this approach generates the reference points and thus forms the reference vectors. The original Das and Dennis's method places points on a normalized hyperplane, where the number of reference points depends on the dimension of objective space *m* and positive integer *H*. The equation can be described as follows:

(2) $$\begin{array}{c} N=\left(\begin{array}{c} H+m-1\\ m-1 \end{array}\right). \end{array}$$

**Figure 1:**
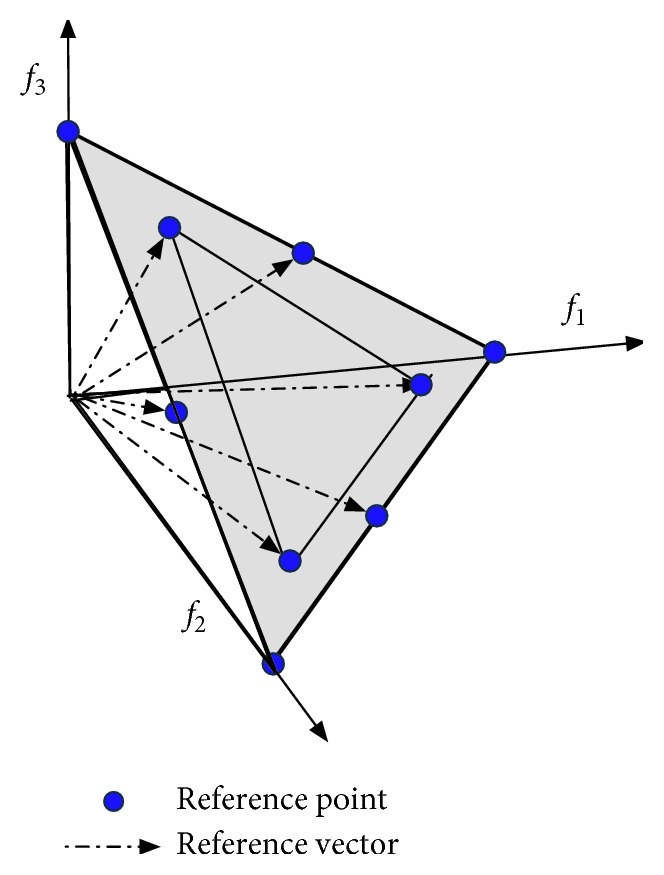
An illustration of the approach to generate the reference point and the reference vector in three objective spaces. As the figure shows, given the *H*_1_=2 and *H*_2_=1, respectively, the final number of the reference point is 9.

However, the number of reference points would rapidly increase when *m* is a relatively large number. To address the drawback of the computational burden of the reference point, a number of new approaches have been proposed [45, 59]. OBEA utilizes the two-layered reference point generation approach, as suggested in [33]. The hyperplane is divided into two parts, which are the boundary and inner layers, as shown in Figure 1. The detail of implementation is as follows:

(3) $$\begin{array}{c} N=\left(\begin{array}{c} H_{1}+m-1\\ m-1 \end{array}\right)+\left(\begin{array}{c} H_{2}+m-1\\ m-1 \end{array}\right). \end{array}$$

The reference vector is the fundamental mechanism for the proposed algorithm. On the one hand, the acute angle and perpendicular distance between the candidate solutions and the reference vectors are adopted in OBEA; on the other hand, the proposed algorithm tends to find near Pareto-optimal solutions corresponding to the reference vectors. Furthermore, the Das and Dennis's systematic approach is utilized in 3-objective and 5-objective problems, while the two-layered reference point generation approach is used in other situations (*M* > 5).

## 3. Proposed Algorithm: OBEA

The pseudocode of the proposed OBEA is presented in Algorithm 1. This algorithm shares a common framework of many evolutionary algorithms and consists of four main phases. First, *N* solutions and reference vectors are initialized with the OBL. Then, potential solutions are selected into the mating pool. In what follows, a set of offspring solutions is obtained by applying crossover and mutation operations. Finally, solution *P* is chosen by adopting the environmental selection procedure. The above steps continue until the termination criteria are met. In the following sections, the details of each component in OBEA will be explained step by step (Algorithm 1).

### 3.1. Initialization with the Opposition-Based Learning

Since the opposition-based learning (OBL) was first proposed by Tizhoosh [60], the OBL has been adopted in various algorithms, which is due to its promising potential to improve the performance of the algorithms [61]. Because of the fact that OBL can obtain fitter starting candidates even when there is no a priori knowledge and enhance the probability of detecting better regions, here we use the OBL to initialize the algorithm. First, the definition of the opposite number and the opposite point is given below.

**Definition 5 .**

Let *x* ∈ [*a*, *b*] be a real number. Its opposite number $\tilde{x}$ is defined as follows [62]:

(4) $$\begin{array}{c} \tilde{x}=a+b-x. \end{array}$$

**Definition 6 .**

Let *x*(*x*_1_, *x*_2_,…, *x*_ *D*_) be a point in *D*-dimensions space, where *x*_*i*_ ∈ [*a*_*i*_, *b*_*i*_], *i*=1,2,…, *D*. Its opposite point $\tilde{x}\left(\overset{˘}{x_{1}},\overset{˘}{x_{2}},\ldots ,\overset{˘}{x_{D}}\right)$ is defined as follows [62]:

(5) $$\begin{array}{c} \tilde{x_{i}}=a_{i}+b_{i}-x_{i}. \end{array}$$

**Figure 2:**
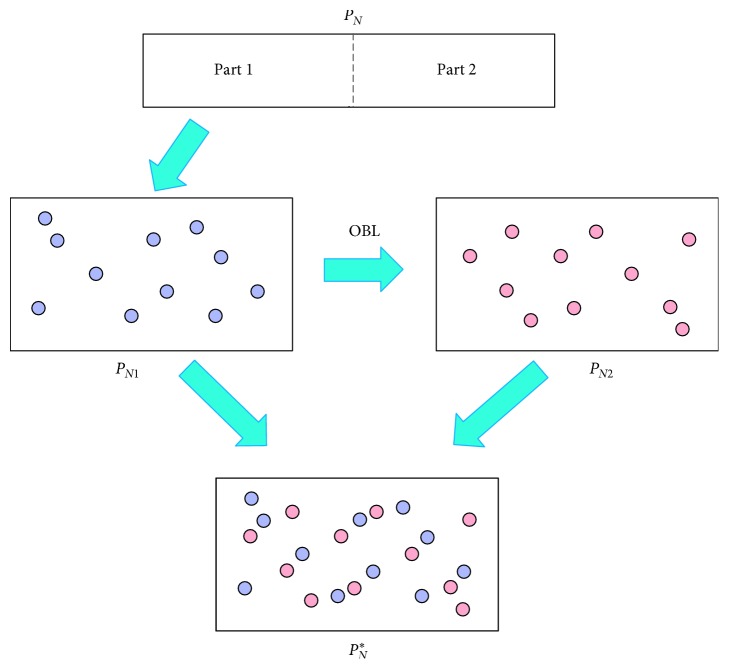
An example showing the initialization phase in OBEA.

The initialization phase of OBEA is shown in Figure 2. Here we first divide population *P*_*N*_ into two parts, and the first half population *P*_*N*1_ is generated by a random distribution. Thereafter, the remaining half population *P*_*N*2_ is initialized in terms of OBL, as follows:

(6) $$\begin{array}{c} P_{N2}=a_{i}+b_{i}-P_{N1}. \end{array}$$

Finally, the set $\left\{\begin{array}{c} P_{N1}{\;\cup }^{}P_{N2} \end{array}\right\}$ is restructured as the initial population *P*_*N*_^*∗*^.

Note that the initialization of OBEA uses the OBL strategy to calculate the opposite point which shares the same idea from our previous work [63]. There are some similarities between them. In detail, both of these algorithms divide the population into two parts and use the random methods and OBL strategy to generate one of them, respectively. Furthermore, both of the algorithms apply the OBL strategy in the process of optimization. However, there are still some differences between our proposed OBEA and our previous work. For our previous work, the OBL strategy-based initialization in this algorithm is only for the multiobjective optimization problem, which the number of the objective is two or three. As for the initialization in our proposed OBEA, the number of objective is far more than that in our previous work as OBEA is designed to solve the many-objective optimization problem. Moreover, OBL strategy in OBEA is also included to design the individual selection and construct the last front with the aim of improving the probability of detecting better regions, which are shown in the Section 3.4. However, OBL strategy in our previous work is only adopted to calculate opposite individuals.

### 3.2. Offspring Creation

In the proposed OBEA, two steps are involved: (1) mating selection, which chooses parents for offspring generation, and (2) variation operation, which generates new candidate solutions. Due to the poor performance to generate offspring solutions in high-dimensional objective space, different methods have been proposed. Here the polynomial mutation and the simulated binary crossover (SBX) [64] are employed as in many other algorithms [59, 65].

### 3.3. Environmental Selection

Environmental selection aims at surviving *N* individuals from the current population and their offspring to select the optimal population with the hybrid selection-based nondominated sort. To be specific, the environmental selection consists of four steps: nondominated sorting, normalization, adaptive clustering, and opposition-based selection. The procedure of environmental selection is shown in Algorithm 2.

#### 3.3.1. Normalization

The normalization procedure incorporated into OBEA is similar to the other algorithms. In normalization, the objective *f*_*i*_(*x*), *i*=1,2,…, *m*, can be replaced as follows:

(7) $$\begin{array}{c} {\overset{ˇ}{f}}_{i}\left(x\right)=\frac{f_{i}\left(x\right)-g_{i}^{\min}}{g_{i}^{\max}-g_{i}^{\min}}, \end{array}$$

where *g*_*i*_^min^ is the ideal point with the aim to find the minimum value of each objective for all solutions in *S*. Similarly, *g*_*i*_^max^ is the nadir point. Moreover, the norm (in the normalized objective space) of each solution in *S* is also calculated, which is used to calculate the acute angle and the perpendicular distance between the candidate solutions and the reference vectors in the normalized objective space. Given *x*_*k*_, *x*_*k*_ ∈ *S*, its norm (denoted as norm(*x*_*k*_)) is defined as follows:

(8) $$\begin{array}{c} \text{norm}\left(x_{k}\right)=\sqrt{\overset{m}{\underset{i=1}{\sum }}{\overset{ˇ}{f}}_{i}{\left(x_{k}\right)}^{2}}. \end{array}$$

#### 3.3.2. Adaptive Clustering Operation

**Figure 3:**
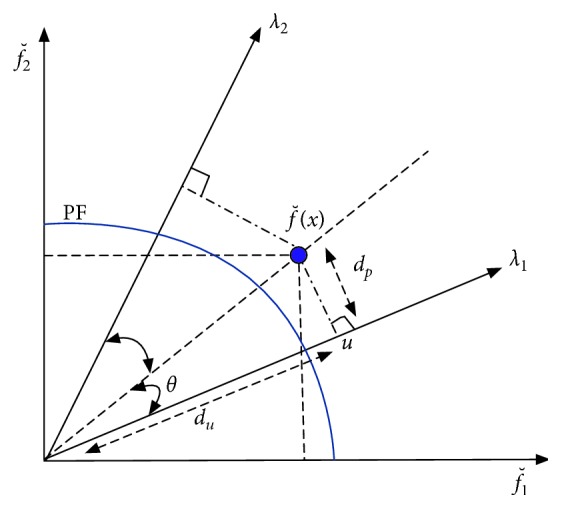
An illustration of the acute angle *θ*, distance *du*, and distance *dp*.

In OBEA, the clustering is done to the population *S* at each generation. After the normalization, population *S* is partitioned into *N* (*N* is the number of reference vectors) sub-populations by associating each individual with its closest reference vector (refer to Figure 3). In the normalized objective space, given the normalized objective vector ${\overset{ˇ}{f}}_{i}\left(x\right)$ and the reference vector *λ*_*j*_, the acute angle between an objective vector and a reference vector can be calculated as

(9) $$\begin{array}{c} \theta_{i,j}=\arccos\frac{{\overset{ˇ}{f}}_{i}\left(x\right)\cdot \lambda_{j}}{\text{norm}\left(x_{i}\right)}. \end{array}$$

Furthermore, *u* is the projection of ${\overset{ˇ}{f}}_{i}\left(x\right)$ on *λ*_*j*_, and the distance between the origin and *u*, denoted as *du*, is calculated as

(10) $$\begin{array}{c} du_{i,j}=\text{norm}\left(x_{i}\right)\cdot \;\cos\;\theta_{i,j}. \end{array}$$

The perpendicular distance between ${\overset{ˇ}{f}}_{i}\left(x\right)$ and *λ*_*j*_, denoted as *dp*, is calculated as

(11) $$\begin{array}{c} dp_{i,j}=\text{norm}\left(x_{i}\right)\cdot \;\sin\;\theta_{i,j}. \end{array}$$

For the clustering operator, as most algorithms only consider *dp* or *θ*, here the sum of the *du* and *θ* with an adaptive mechanism will be involved together as

(12) $$\begin{array}{c} {\text{AD}}_{i,j}=\left(1-s\right)\cdot du_{i,j}+s\cdot \theta_{i,j}, \end{array}$$

where *s* is a sigmoid function which can be described as follows:

(13) $$\begin{array}{c} s=\frac{1}{1+\exp\left(\left(\mu -\left(t/t_{\max}\right)\right)\times 15\right)}, \end{array}$$

where *t* is the current generation number, *t*_max_ is the maximal generation number, and *μ* ∈ [0,1) is the control parameter.

As Algorithm 3 shows, the core concept of the adaptive clustering operation is to allocate individuals to the subpopulations with the AD values. To be more specific, for one individual *S*_*i*_, we first calculate the AD_*i*,*j*_ between the individual and each reference vector. Thereafter, minimum AD is obtained; thus, the related subpopulation *C*_*k*_ is the cluster where the individual *S*_*i*_ belongs to. In this way, an individual *S*_*i*_ is allocated to a subpopulation *C*_*k*_ if and only if the AD_*i*,*j*_ is minimal.

### 3.4. Opposition-Based Selection

To solve the MOPs with good convergence and diversity, the opposition-based selection has been conducted. The main idea of the proposed opposition-based selection is taking the advantages of opposition-based learning and combined the clustering operation into an efficient method to achieve the goal. Following paragraph will describe the selection mechanism in the details, and the general framework is shown in Algorithm 4.

In the opposition-based selection, we first construct the last front *F*_l_ through the cluster *C*. To be more specific, our motivation is to find the solution on each reference vector that is closest to the ideal point with the better convergence criterion and diversity. Hence, a hybrid distance (HD) is proposed where the acute angle and perpendicular distance between the candidate solutions and the reference vectors are combined through an adaptive mechanism to select the candidate in the cluster *C* effectively. The hybrid distance is as follows:

(14) $$\begin{array}{c} {\text{HD}}_{i,j}=\left(1+\frac{k}{K}\;*\;\gamma \right)\cdot \left\|{\overset{ˇ}{f}}_{i}\right\|, \end{array}$$

where *k* is the current generation number and *K* is the maximal generation number. *γ* can be described as *γ*=(*dp*_*i*,*j*_/*du*_*i*,*j*_)+(*θ*_*i*,*j*_/Θ_*i*,*j*_), and the Θ_*i*,*j*_ is the smallest angle value between the reference vector and the other reference vectors in the current generation.

After constructing the last front *F*_l_, we calculate the opposite value of individuals in *F*_l_ according to the OBL in Section 3.1. Whereafter, the opposite values are added into *F*_l_. Finally, the *K* individuals are randomly selected from *F*_l_ to construct *P*_next_.

### 3.5. Computational Complexity of OBEA

The normalization and the calculation of norm in OBEA require *O*(*mN*) additions. The time complexity for clustering is *O*(*mN*^2^). In addition, the selection holds a computational complexity of *O*(*mN*^2^). To sum up, the overall worst complexity of one generation of OBEA is approximately *O*(*mN*^2^).

## 4. Experiment Description

In this section, experiments with twelve algorithms such as MOEA/D, dMOPSO [66], MOMBI2 [67], *ϵ*-MOEA [68], NSGA-III, RVEA, MaOEARD [69], MyODEMR [70] MOEA/DD, MOEA/DVA [71], Two_Arch2, and SPEAR have been adopted in order to evaluate the performance of the proposed OBEA algorithm on 15 benchmark test problems taken from two widely used DTLZ test suites [72] and WFG test suites [73]. For each test problem, objective numbers varying from 3 to 15, i.e., *M* ∈ {3,5,8,10,15}, are considered.

In the following subsections, the test problems and the quality indicators used in our comparative experiments are first presented. Then, the experimental settings adopted in this study are provided. Moreover, thirty independent runs are executed for each test problem to avoid randomness, and the Wilcoxon rank sum test is adopted to compare the results obtained by OBEA and those nine compared algorithms at a significance level of 0.05.

### 4.1. Test Problems

Aiming to evaluate the performance effectively, two well-known test suites Deb-Thiele-Laumanns-Zitzler (DTLZ) and Walking-Fish-Group (WFG) are involved in the experiments, as shown in Table 1. Since the nature of DTLZ5 and DTLZ6s PFs is unclear beyond three objectives [73], here we only consider DTLZ 1–4 and DTLZ7 problems for the DTLZ test suite. The main features of these problems are summarized in Table 2. For DTLZ1-DTLZ4 and DTLZ7, the total number of decision variables is given by *n*=*m*+*k* − 1, where *m* is the number of objectives and *k* is set to 5 for DTLZ1, 10 for DTLZ2-DTLZ4, and 20 for DTLZ7. As for all WFG problems, the number of decision variables is set to 24 and the position-related parameter is set to *m* − 1 according to [33, 73].

### 4.2. Performance Metrics

In our experimental study, two widely used metrics are chosen to evaluate the performance of each algorithm, which are named the Inverted Generational Distance (IGD) [74] and Hypervolume (HV) [75]. The IGD and HV can measure both the convergence and diversity of obtained solutions effectively.

#### 4.2.1. Inverted Generational Distance (IGD)

Let *P*^*∗*^ denote a set of uniformly distributed solutions in the objective space along the Pareto front. *P* is an approximation to the PF, which is obtained by the algorithm. The IGD is described as

(15) $$\begin{array}{c} \text{IGD}\left(P,P^{*}\right)=\frac{\sum_{\left|P^{*}\right|}^{i=1}\text{dist}\left(P_{i}^{*},P\right)}{\left|P^{*}\right|}, \end{array}$$

where dist(*P*_*i*_^*∗*^, *P*) is the Euclidean distance between a point *x*^*∗*^ ∈ *P*^*∗*^ and its nearest neighbor in *P*, and |*P*^*∗*^| is the cardinality of *P*^*∗*^. It can be seen from the definition of IGD that, for a large |*P*^*∗*^|, it can cover approximately the entire Pareto front, which is another aspect of metric in terms of diversity.

#### 4.2.2. Hypervolume (HV)

Consider the set of final nondominated points *S* and a reference point *a*=(*a*_1_, *a*_2_,…,*a*_*m*_)^*T*^ in the objective space which is dominated by any point in the set *S*. Then, the hypervolume of *S* with regard to *a* can be described as follows:

(16) $$\begin{array}{c} \text{HV}\left(S,a\right)=\text{volume}\left(\underset{f\in A}{\cup }\left[f_{1},a_{1}\right]\times \cdots \left[f_{m},a_{m}\right]\right). \end{array}$$

It should be noted that choosing *r* that is slightly larger than the nadir point *g*_nad_ is suitable [76, 77]. Here, we set *a* to 1.1*g*_nad_, which is the same as in [33]. In addition, for problems with no more than 10 objectives, the recently proposed fast hypervolume calculation method is adopted to calculate the exact hypervolume [78]. As for problems having 15 objectives, the Monte Carlo method [37] with 1,000,000 sampling points is adopted, and all hypervolume values presented in this work are all normalized to [0,1].

### 4.3. Parameter Settings

As for the parameter settings, several general settings for algorithms are given as follows:

1. *Population Size.* The setting of the population size *N* for NSGA-III, MOEA/D, MOEA/DD, MOEA/DVA, and OBEA is controlled by a parameter *H.* Since MOMBI2 involves a binary tournament selection, we use the same population size as in NSGA-III or OBEA. Moreover, for the other algorithms, the population size is the same as above for ensuring a fair comparison. Population sizes *N* used in this study for different number of objectives are listed in Table 3.
2. *Parameters for Operator.* Since the PBI function is involved in MOEA/D, dMOPSO, Two_Arch2, and MOEA/DD, the penalty parameter *θ* is set to 5 as suggested in [43]. Furthermore, as the simulated binary crossover (SBX) and polynomial mutation are employed to generate offspring solutions, the crossover probability *p*_c_ and mutation probability *p*_m_ are set to 1.0 and 1/*n*, respectively. For the SBX operator, its distribution index is *η*_c_  = 30, and the distribution index of the mutation operator is *η*_m_  = 20 [45].
3. *Parameter Settings for Algorithms.* Besides the parameters mentioned above, algorithms also have their specific parameters. These parameters are set mainly according to the suggestions given by their developers with the purpose of ensuring the impartiality and objectivity of the experiment. The details are shown below.
  - Parameter setting in MOEA/D: the neighborhood size *T* is set to 20 [43].
  - Parameter setting in MOEA/DD: the neighborhood size *T* is set to 20, and the probability used to select in the neighborhood is *δ*=0.9[45].
  - Parameter setting in MOEA/DVA: the neighborhood size *T* is set to 20, the number of sampling solutions in control variable analysis is 20, and the maximum number of tries required to judge the interaction is 6 [71].
  - Parameter setting in dMOPSO: the age threshold *T*_a_ is set to 2 [66].
  - Parameter setting in MOMBI2: following the practice in [67], two parameters in MOMBI2 are set as *ε* =  1*e* − 3 and *α* = 0.5, respectively [67].
  - Parameter setting in *ϵ*-MOEA: the parameter in grid location calculation is *ϵ*=0.06[68].
  - Parameter setting in RVEA: in the experimental comparisons, *α* = 2 and *f*_*r*_  = 0.1 are used for all test instances [53].
  - Parameter setting in Two_Arch2: the CA size is equal to the population size, and the fractional distance *p* is equal to 1/*m* (where *m* is the number of the objective) [49].
  - Parameter setting in OBEA: *μ* = 0.35 is used for the adaptive clustering mechanism.

### 4.4. Experimental Results and Analysis

In this section, OBEA is compared with the four efficient evolutionary algorithms that are proposed in Section 4.4.1. Then, six state-of-the-art evolutionary algorithms are also included as the comparators to investigate the ability of OBEA for solving many-objective optimizations in Section 4.4.2. The analysis of the adaptive strategy design is included in Section 4.4.3, and the discussion of the effectiveness analysis of OBL and adaptive strategy is present in Section 4.4.5, followed by the comparisons between HD, TCH, and PBI in Section 4.4.4. Furthermore, investigation of the evolutionary behavior of OBEA on parts of test problems is also included in Section 4.4.6.

#### 4.4.1. Comparison with the Previous Algorithms

Statistical results of the IGD values obtained by OBEA and four algorithms named MOEA/D, dMOPSO, MOMBI2, and *ϵ*-MOEA are summarized in Tables 4 and 5, where the best results are italicized. The significance of difference between OBEA and the peer algorithms is determined by using the Wilcoxon rank sum test, where “+,” “−,” and “=” indicate the competitor is better than, worse than, or similar to the proposed OBEA, respectively, and the results are summarized as “*w*/*l*,” which denotes that corresponding competitor wins on *w* functions, loses on *l* functions, and ties on *t* functions, compared with the proposed OBEA.

It can be observed that OBEA covers the majority of the best values among the compared algorithms on the five original DTLZ test instances, especially on DTLZ1, DTZL3, and DTLZ7. As for the DTLZ2 test problem, the best values on 5-objective, 8-objective, and 10-objective are obtained by *ϵ*-MOEA and MOEA/D, respectively. However, the proposed OBEA tends to perform well on 3-objective and 15-objective test instances. While, for the DTLZ4 test problem, the performance of OBEA is little worse than MOMBI2 and *ϵ*-MOEA on 3-objective, 10-objective, and 15-objective instances, the proposed algorithm still outperforms the others on 5-objective instance and is similar to *ϵ*-MOEA on the 8-objective test instance according to the Wilcoxon rank sum test.

The statistical results in Table 5 also indicate that OBEA has achieved the best performance among the five algorithms on all WFG1 and WFG6–9 test instances. For WFG2 and WFG3 test problems, *ϵ*-MOEA and MOMBI2 show best performance on most test instances. However, the proposed OBEA tends to perform well on parts of instances. Furthermore, OBEA still outperforms the other algorithms like MOEA/D and dMOPSO on most instances according to the Wilcoxon rank sum test. As for the WFG4 and WFG5 test instances, *ϵ*-MOEA and OBEA cover all the best values among five algorithms, which also verify the best performance of the proposed algorithm.

As evidenced by statistical results of the HV values on DTLZ test suits summarized in Table 1, OBEA has covered the best values on DTLZ1 and DTLZ3 test problems. As for DTLZ2, the best values on 8-, 10-, and 15-objective instances have been obtained by OBEA, while MOMBI2 and *ϵ*-MOEA have achieved the best performance on different instances, respectively. Moreover, the best values on the 15-objective DTLZ4 test instance have been obtained by OBEA, which indicates the best performance of the proposed algorithm for high-dimensional problems. By contrast, the performance of OBEA on the DTLZ7 test functions is not as good as that on the DTLZ1 and DTLZ3 test functions; however, the performance of the proposed algorithm is superior to MOEA/D and *ϵ*-MOEA on most DTLZ7 test instances.

Similar observations can be made about the results on the WFG test problem, where OBEA has shown the most competitive performance on most test instances in Table 6, while MOMBI2 and *ϵ*-MOEA have also achieved the best performance on different instances, respectively. Above all, it can be concluded that OBEA can balance the diversity and convergence better than these four algorithms on most instances.

#### 4.4.2. Comparison with the State-of-the-Art Algorithms

In this section, the proposed algorithm is compared with several state-of-the-art algorithms on the DTLZ and WFG test problems. The experimental results of each compared algorithms on the DTLZ and WFG test instances are shown in Tables 7 and 8, in terms of IGD metrics. The experimental results of HV on these instances are also demonstrated in Tables 9 and 10. Moreover, thirty independent runs are executed for these algorithms on the test problems with the Wilcoxon rank sum test at a significance level of 0.05.

It can be observed that OBEA obtained most of the best values among the compared algorithms on the five original DTLZ test instances. For DTLZ1, OBEA and MOEA/DVA show better performance than the other algorithms according to the Wilcoxon rank sum test. For the DTLZ2 and DTLZ7 test problems, although Two_Arch2 and MOEA/DD, together with MOEA/DVA, cover parts of the best values, OBEA still tends to outperform most algorithms on these instances. As for the DTLZ3, although NSGA-III and RVEA share the same idea with OBEA in using the reference line, OBEA is still superior to NSGA-III and RVEA, which may due to the effectiveness of selection strategy in our algorithm. As for the DTLZ7, MOEA/DD, Two_Arch2 and MOEA/DVA shows good performance on parts of instances. However, the proposed OBEA shows competitive results on the other high-dimensional instances.

As for IGD results on WFG test problems shown in Table 8, OBEA still perform well on most test instances. To be more specific, OBEA covers all the best values on WFG1 and WFG7 test problems, which shows the best performance of the proposed algorithms. As for the WFG2 test problem, which covers the convex, disconnected, and nonseparable PF, the performance of OBEA is a little worse than the NSGA-III and Two_Arch2. However, the proposed OBEA still tends to outperform the other algorithms on parts of instances in WFG2. Although the best values on 3-objective, 8-objective, and 10-objective instances in WFG3 are obtained by MOEA/DVA and Two_Arche2, respectively, OBEA covers all the other instances on the WFG3 test problem. For the WFG4 test problem, OBEA and MOEA/DVA, together with Two_Arch2, obtain the best value. As for the WFG5 and WFG6 test problems, although RVEA covers the best value on 10-objective instance, OBEA still tends to outperform most of the algorithms on the remaining instances. Although best values on some instances of WFG8 have been covered by MOEA/DD and Two_Arch2, OBEA still obtained the best performance on 5-objective and 8-objective test instances. As for the WFG9, NSGA-III shows better performance than that on the DTLZ test problem. However, OBEA still outperforms most of the algorithms, especially on 3-objective, 5-objective, and 15-objective test instances of WFG9.

The statistical results of the HV values obtained by the six algorithms are summarized in Tables 9 and 10. The values of HV presented in tables are all normalized to [0,1]. Clearly, a higher value of HV is recommended. OBEA obtains the majority of the best median values over the instances on DTLZ, which indicates the obvious advantages of the proposed algorithm. For the DTLZ1 test problem, although MOEA/DVA obtains the best values on 8-objective and 10-objective instances, OBEA shows good performance on 3-objective, 8-objective, and 15-objective instances. Since DTLZ2 test problems involve concave PFs, Two_arch2 and MOEA/DVA, together with RVEA, obtain the best performance on 3-objective, 8-objective, and 15-objective instances, respectively, while OBEA covers the best values on 5-objective as well as 10-objective instance and tends to be far more superior to the other algorithms. Furthermore, although DTLZ3 is a concave and multimodal test problem, OBEA obtains most of the best HV value on this test problem. As for the DTLZ4, the performance of OBEA is little worse than Two_Arch2 and MOEA/DVA. However, OBEA still outperform most of the algorithms, especially on 15-objective DTLZ4. In addition, due to the fact that disconnected and multimodal PFs have been employed in the DTLZ7 test problem, the performance of OBEA is not that good on 5-objective and 8-objective instances; however, OBEA still outperforms other algorithms, especially on the other instances.

**Figure 4:**
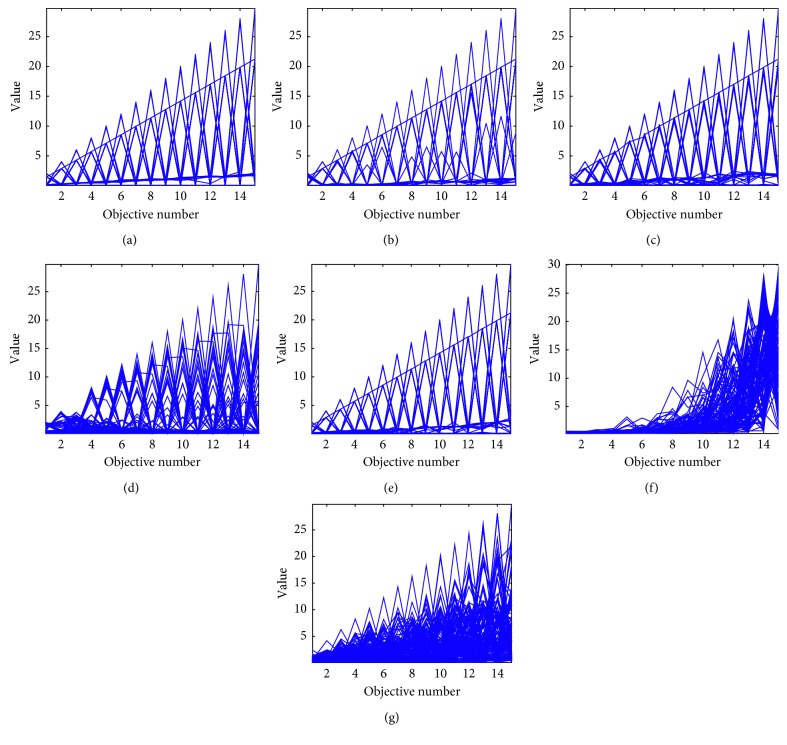
Parallel coordinates of the nondominated front obtained by each algorithm on 15-objective WG5 in the run associated with the median HV value. (a) OBEA on WFG5. (b) RVEA on WFG5. (c) NSGA-III on WFG5. (d) MOEA/DD on WFG5. (e) SPEAR on WFG5. (f) MOEA/DVA on WFG5. (g) Two Arch2 on WFG5.

Statistical results of the HV values on WFG test sets are shown in Table 10. OBEA has shown the most competitive performance on WFG1, WFG6, and WFG8. By contrast, performance of RVEA on the WFG test functions is not as good as that on the DTLZ test functions. However, SPEAR shows good performance on WFG test problems, especially on the WFG5 test problem. Moreover, although the performance of OBEA is little worse than Two_Arch2 on some instances in WFG2, it still tends to be superior on most test instances like the 15-objective WFG5 instance, which is shown in Figure 4. As for the WFG2 test problem, the performance of NSGA-III is much better than that on other test instances. Finally, for the WFG7 and WFG9 test problems, although Two_Arch2 and MOEA/DVA cover some of the best values, OBEA still shows the best performance among most algorithms on these test instances.

In a word, OBEA can obtain the best performance on these test problems from multiobjective to many-objective. This may be due to the fact that the exploration ability and convergence of OBEA are emphasized due to the employed OBL and adaptive clustering mechanisms, which lead the results revealing high efficiency. Note that the performance of OBEA may become little worse while handling some test problems with irregular PF. This is because OBEA uses a set of uniformly distributed reference vectors to handle the problem. While using the uniformly distributed reference vectors is based on an assumption that the PF has a regular geometrical structure, this may have the influence on the performance of OBEA on the test problem with irregular PF. To sum up, the proposed OBEA can obtain the best performance on most test problems with the PF which has regular geometrical structure. As for the problem with quite irregular PF, the performance of OBEA is not as well as on the previous problem. However, the proposed OBEA still tends to provide the competitive results.

As to quantify how well each algorithm performs with the HV overall, the performance score [37] is introduced to rank all the algorithms. Given a specific problem instance, suppose there are *k* algorithms Alg_1_, Alg_2_,…, Alg_*k*_ involved in the comparison. *δ*_*i*,*j*_ is set to 1 if Alg_*j*_ is significantly better than Alg_*i*_ in terms of HV, and 0 otherwise. Thereafter, for each algorithm Alg_*i*_, the performance score *P*(Alg_*i*_) is determined as follows:

(17) $$\begin{array}{c} P\left({\text{Alg}}_{i}\right)=\overset{k}{\underset{j=1,j\neq i}{\sum }}\delta_{i,j}. \end{array}$$

**Figure 5:**
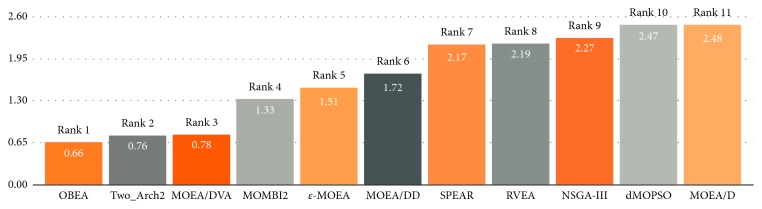
Ranking and score of average performance obtained by each compared algorithms in terms of HV.

This value represents the number of algorithms which have significantly better performance than the corresponding algorithm on the entire tested instances, and zero means that no other algorithm tends to be significantly better in terms of the HV indicator. Clearly, the smaller the index, the better the algorithm; Figure 5 shows the average performance score over all 70 test instances among the ten algorithms in terms of HV, and the rank of each algorithm according to the score is also given.

**Figure 6:**
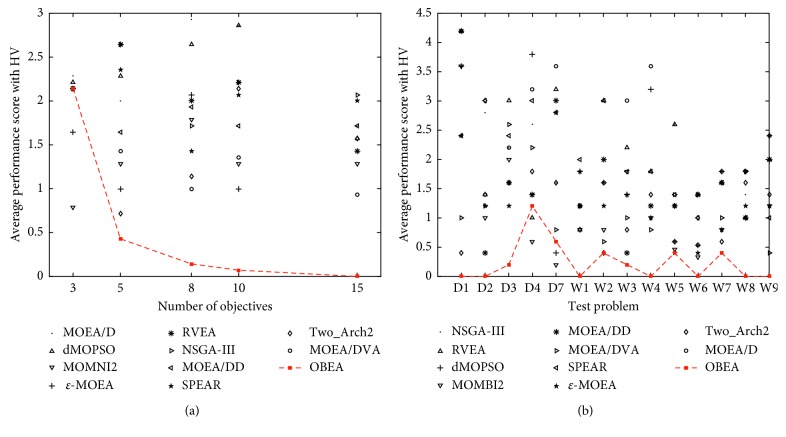
(a) Average performance score obtained by eleven algorithms over all test problems of different numbers of objectives in terms of the HV and (b) average performance score obtained by ten algorithms on dimensions for different test problems in terms of the HV, Dx for DTLZ, and Wx for WFG. The values of the proposed OBEA are connected by a solid red line.

As the figure shows, OBEA is in the first place among the eleven algorithms, which indicates the better performance of the proposed algorithm on these test problems. Note that although MOEA/D has been ranked in the last place, it still shows good performance on some test problems. Moreover, in order to better visualize the performance, the average performance score summarized for different numbers of objectives and different test problems in terms of the HV are presented in Figure 6, respectively.

Figure 6(a) shows the average performance over all test problems for different number of objectives. The proposed OBEA works well on nearly all the considered problems of diverse objectives expect on 3-objective problems. However, OBEA still outperforms most of the algorithms on 3-objective problems. Furthermore, the performance score for the individual test problems is shown in Figure 6(b), which covers all number of objectives. For DTLZ1-DTLZ3, OBEA shows good performance and nearly outperforms all other algorithms. As for DTLZ4 and DTLZ7, the performance of OBEA is not as good as that on DTLZ1-3 but still is generally superior among the corresponding algorithms. At last, for the WFG test problem, OBEA nearly covers the best values. In a word, OBEA is under a good performance among the ten algorithms in terms of the HV.

#### 4.4.3. Analysis of Adaptive Clustering Mechanism

In the proposed OBEA, the adaptive clustering is adopted to assist the algorithm for selecting the individual from each subpopulation. Nowadays, different approaches have been proposed to cluster the individuals. For example, RVEA and NSGA-III use the angle to partition the population and *θ*-DEA uses the perpendicular distance to cluster the individuals. Although most of these approaches simply focus on the acute angle or distance alone to cluster the individuals and may have superior performance in some situations. However, simplex use of the acute angle or distance may lead the crowding of individuals in cluster increase and lose some better situations and thus affects the performance of the algorithm in balancing diversity and convergence.

**Figure 7:**
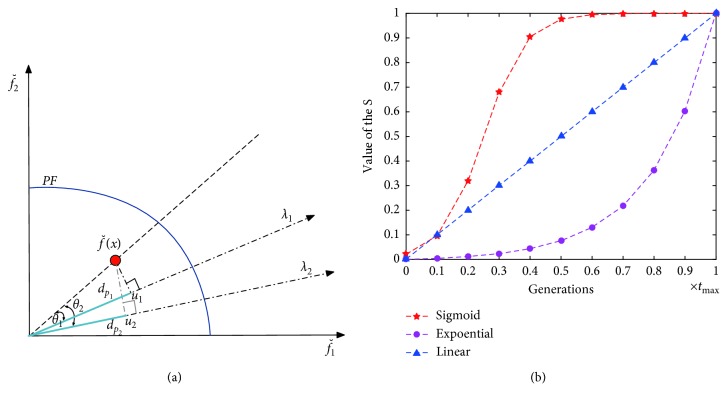
(a) An illustration of the special situation when solely using the acute angle or perpendicular distance to select individual. (b) An illustration of the three functions versus the iterative generations.

In the view of this, in our proposed OBEA, the acute angle and the distance are considered comprehensively with an adaptive mechanism to assist the algorithm to assign individuals efficiently. For example, in Figure 7(a), giving the individual *x* and its normalization $\overset{ˇ}{f}\left(x\right)$, *λ*_1_ and *λ*_2_ are two different reference vectors. *θ*_1_ and *θ*_2_ indicate the acute angles between $\overset{ˇ}{f}\left(x\right)$ and *λ*_1_ and *λ*_2_, respectively. Moreover, *d*_*p*1_ and *d*_*p*2_ indicate the distances between the origin and the projection of $\overset{ˇ}{f}\left(x\right)$ on *λ*_1_ and *λ*_2_, respectively. Considering the distance-based approach to assign the individual *x*, the reference vector *θ*_2_ will be the preferred one as its distance *d*_*p*2_ is smaller than the distance *d*_*p*1_. However, to focus on the acute angle-based clustering, the individual *x* will be associated with *λ*_1_ according to the fact that the acute angle of *θ*_1_ is smaller than the angle of *θ*_2_, obviously. In our adaptive clustering strategy, the individuals will be associated with the reference vector according to the distance between the origin and the projection point at first to increase the diversity of the individuals in the cluster. Thereafter, with increase in the number of iterations, the individuals will be assigned according to the angle through our adaptive function.

As the function *s* is the key point in the adaptive scheme, here we consider the following three different functions to do the further analysis:

(18) $$\begin{array}{c} \mathrm{linear}:\;s=\left[\frac{t}{t_{\max}}\right],\\ \mathrm{sigmoid}:\;s=\left[\frac{1}{1+\exp\left(\left(\mu -t/t_{\max}\right)\times 15\right)}\right],\\ \mathrm{exponential}:\;s=\left[\frac{\exp\left(5t/t_{\max}\right)-1}{\exp\left(5\right)-1}\right]. \end{array}$$

**Figure 8:**
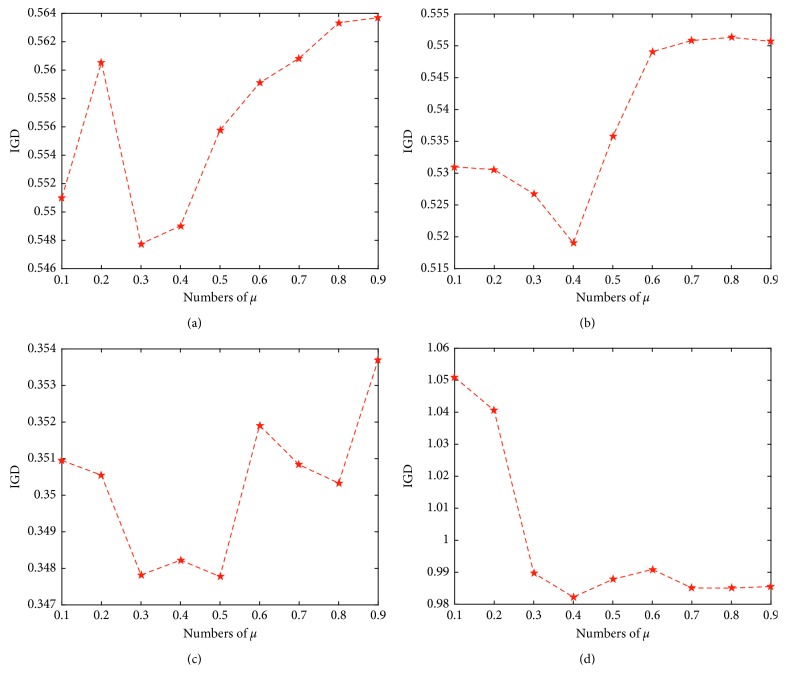
(a) An illustration of the IGD results on 15-objective DTLZ2 when using different *μ*. (b) An illustration of the IGD results on 10-objective WFG7 when using different *μ*. (c) An illustration of the IGD results on 8-objective DTLZ4 when using different *μ*. (d) An illustration of the IGD results on 5-objective WFG4 when using different *μ*.

Here, Figure 7(b) shows the values of *S* using three different functions. *t* is the current generation number, *t*_max_ is the maximal generation number, and [·] is the ceiling function. Moreover, *μ* is the phase control parameter for the sigmoid function. As Figure 8 shows, the IGD results on different test problems when using various *μ* values have indicated that 0.3, 0.4 is the best setting for the algorithm. Due to the fact that the function can obtain the standard phase in [0, 1] with this value of *μ* equal to 0.3 or 0.4 as shown in Figure 8, here *μ*=0.35 is adopted in the following experiments. In order to validate the effectiveness of the proposed schemes, we compare OBEA (here we named our algorithm OBEA-sig) with the following two variants.

1. Variant I: in this variant, the adaptive scheme in OBEA is implemented based on adaptive clustering with the linear function (OBEA-lin).
2. Variant II: the difference of this variant and OBEA is that the adaptive scheme is constructed through the exponential function (OBEA-exp).

In this study, OBEA with two variants are tested on the DTLZ test suite and the WFG test suit with three to fifteen objectives for 30 runs and applied HV to quantify the performance. The parameter settings of the two variants are the same as in OBEA. The mean and standard deviation of HV values of the two variants as well as OBEA are listed in Table 11, where the best mean value for each test instance is italicized. Also, the Wilcoxon rank sum test is performed to investigate whether there is a significant performance difference between OBEA and its two variants. As Table 11 shows, OBEA-sig obtains most of the best mean values among the DTLZ and WFG test instances. Although OBEA-lin and OBEA-exp have obtained parts of the best values, OBEA-sig still performs well on most test instances according to the Wilcoxon rank sum test. In a word, OBEA-sig is more stable and efficient than others. Therefore, the sigmoid function of adaptive strategy is adopted in this paper.

#### 4.4.4. Analysis of Opposition-Based Selection

The key point in our opposition-based selection part is the proposed hybrid distance (HD). Here, we use the contrast experiment to further explore the effectiveness of the strategy. Note that the formulation of HD shares some similarity to angle-penalized distance (APD) [53] and penalty-based boundary intersection (PBI) [43], which are adopted in the RVEA and decomposition-based MOEAs, respectively. In this section, the comparative studies on the original OBEA, our proposed algorithm with the APD approach, and the proposed algorithm with the PBI approach are carried out. In particular, we replace the HD with APD or PBI. Thereafter, the opposition point is calculated according to the Section 3.4. Moreover, the experiment is also conducted with the Wilcoxon rank sum test. For simplicity, the proposed algorithm with the APD and PBI approaches are denoted as OBEA-APD and OBEA-PBI, respectively.

The performance of OBEA, OBEA-APD, and OBEA-PBI has been verified on four benchmark test problems selected from different test suites named DTLZ1, DTLZ2, WFG2, and WFG4. As shown by the statistical results summarized in Table 12, OBEA shows the best performance on most problems, especially for the DTLZ2, on which the best values are all obtained by the proposed algorithm with HD. As for OBEA-APD and OBEA-PBI, these two algorithms obtain the best values on some instances. However, performance of OBEA on these instances is equal to these two algorithms according to the Wilcoxon rank sum test. To sum up, the results indicate that the proposed HD has better capability and effectiveness than APD and PBI, which further verify the effect of the proposed strategy on handling the many-objective problems.

#### 4.4.5. Effectiveness Analysis of OBL and Adaptive Clustering in OBEA

As two main strategies have been designed in our OBEA, here three variational algorithms are generated to investigate the effectiveness of each strategy. In particular, they are OBEA1 (OBEA without OBL strategy), OBEA2 (OBEA without adaptive clustering strategy), and OBEA3 (OBEA without OBL strategy and adaptive clustering strategy). Moreover, the performance of these algorithms is shown in Table 13 and the better values are italicized.

The results show that the two strategies both have ability to improve the performance of the algorithm. In detail, although OBEA1 and OBEA2 both show the better capability of searching for the best function values for most cases, the performance of OBEA2 is slightly worse than OBEA1 according to the Wilcoxon rank sum test, which indicates that adaptive strategy has further influence on assisting algorithm than the OBL strategy. This can be attributed to the best structure of the adaptive clustering mechanism and the effectiveness of the reference line. Moreover, although the performance of OBEA3 is little worse than the others, the values of the HV of this variation still tend to be good, which indicates the best potential of nondominated sorting in OBEA. To sum up, due to good performance of these strategies, our proposed OBEA gains best potential in solving different kinds of problems.

#### 4.4.6. Investigation of the Evolutionary Behavior

**Figure 9:**
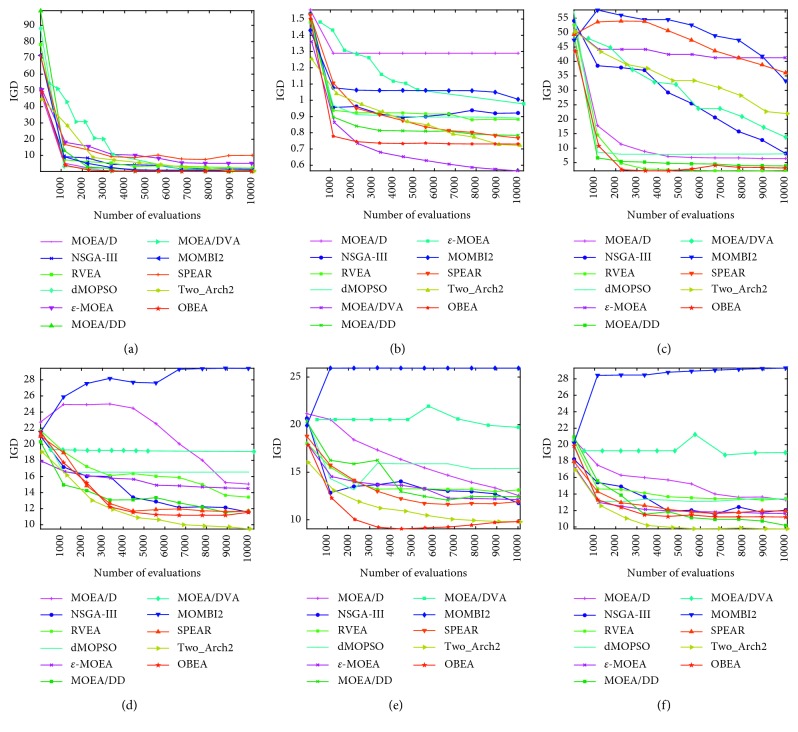
Trajectory of the mean IGD value on ten algorithms with fifteen objectives. (a) DTLZ1. (b) DTLZ4. (c) DTLZ7. (d) WFG4. (e) WFG7. (f) WFG9.

As to observe the evolutionary behaviors of the algorithms, further studies with these ten algorithms have been carried out to exhibit the evolutionary trajectories. Moreover, Figure 9 plots the performance trajectories of IGD versus the number of function evaluations for the ten algorithms on the 15-objective DTLZ1, DTLZ4, DTLZ7, WFG4, WFG7, and WFG9 test instances. Depending on the figures, OBEA shows an obvious advantage over most of its competitors. Although the performance of OBEA is little worse on DTLZ4, the figure clearly shows that the proposed OBEA still tends to be superior to the other algorithms on these two instances. By simultaneously considering the IGD, HV, and evolutionary behaviors, we can conclude that the overall performance of OBEA is better than or at least equal to the nine competitors.

## 5. Conclusion

Due to the loss of selection pressure and the ineffective design of balancing the diversity and convergence, general MOEAs always encounter challenges in solving MaOPs. Although diversity of algorithms, especially the reference-based algorithms, have been designed to address this issue, most algorithms that belong to these categories simply use the angle or distance solely to measure the quality of the population members with the reference set, which may lose some good solutions due to their simplex selection mechanism. Therefore, we have presented a new many-objective evolutionary algorithm, called OBEA. Two strategies called OBL and adaptive clustering have been adopted in environmental selection with the purpose of improving the performance of the algorithm in balancing the convergence and the diversity.

To establish the strong competitiveness, we have employed extensive experimental comparison of OBEA with ten algorithms. A number of well-known benchmark problems such as DTLZ test suits and WFG test suits are chosen to explore the abilities of the algorithms. The statistical results reveal that the proposed OBEA performs well on almost all the problem instances, and it obviously outperforms most state-of-the-art many-objective optimizers. However, the result also indicates that none of the algorithms is capable of preceding any of the other algorithms on all the instances which reflect the importance of the choice of algorithms carefully when solving the MaOPs.

In the future, we will extend OBEA to solve constrained many-objective problems by incorporating constraint handling techniques in order to further verify its effectiveness.

## Figures and Tables

**Algorithm 1:**
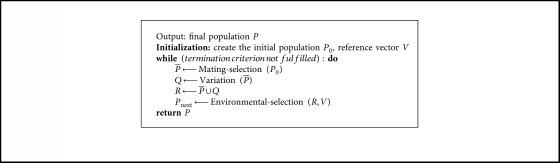
General framework of OBEA.

**Algorithm 2:**
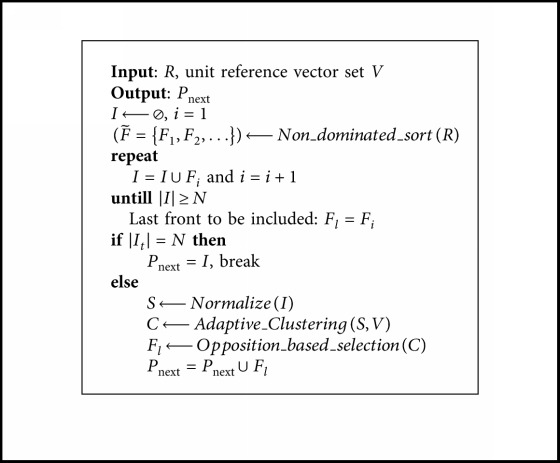
Environmental selection.

**Algorithm 3:**
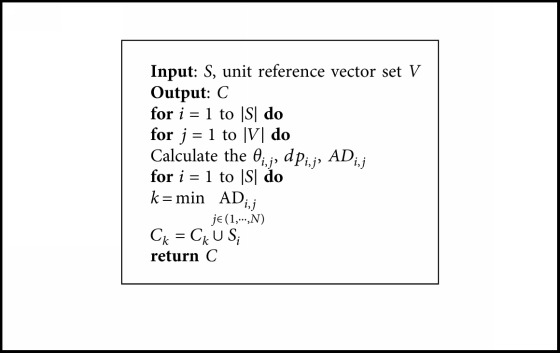
Adaptive clustering operation.

**Algorithm 4:**
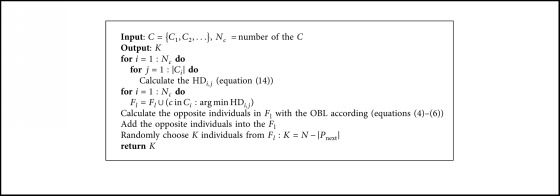
Opposition-based selection.

**Table 1:** The statistical results (mean and standard deviation) of the HV values obtained by each algorithm on DTLZ1 to DTLZ4 and DTLZ7. The best results are italicized.

Problem	M	MOEAD	dMOPSO	MOMBI2	*ϵ*-MOEA	OBEA
DTLZ1	3	3.994*e* − 1 (3.36*e* − 1) −	7.015*e* − 3 (3.84*e* − 2) −	5.819*e* − 1 (3.01*e* − 1) −	2.931*e* − 1 (2.89*e* − 1) −	*8.471e* *−* *1 (6.13e* *−* *3)*
	5	5.010*e* − 1 (3.49*e* − 1) −	2.856*e* − 4 (1.56*e* − 3) −	1.406*e* − 1 (2.68*e* − 1) −	8.164*e* − 2 (2.00*e* − 1) =	*9.761e* *−* *1 (4.15e* *−* *4)*
	8	5.824*e* − 1 (2.20*e* − 1) −	5.418*e* − 3 (2.51*e* − 2) −	2.923*e* − 1 (3.37*e* − 1) −	3.102*e* − 1 (5.34*e* − 3) −	*9.524e* *−* *1 (6.84e* *−* *3)*
	10	4.907*e* − 1 (2.76*e* − 1) −	1.031*e* − 2 (2.31*e* − 2) −	9.039*e* − 1 (9.79*e* − 2) =	8.356*e* − 2 (1.53*e* − 1) −	*9.571e* *−* *1 (4.18e* *−* *3)*
	15	3.566*e* − 1 (2.33*e* − 1) −	5.092*e* − 3 (1.76*e* − 2) −	2.195*e* − 1 (2.87*e* − 1) −	4.015*e* − 1 (6.12*e* − 2) −	*9.456e* *−* *1 (4.46e* *−* *6)*

DTLZ2	3	5.551*e* − 1 (7.76*e* − 4) −	3.671*e* − 1 (1.60*e* − 2) −	*5.572e* *−* *1 (5.51e* *−* *4)* +	5.409*e* − 1 (2.93*e* − 3) −	5.563*e* − 1 (5.70*e* − 4)
	5	7.823*e* − 1 (5.77*e* − 3) =	4.986*e* − 1 (1.69*e* − 2) −	7.881*e* − 1 (3.38*e* − 3) =	*8.022e* *−* *1 (5.35e* *−* *3)* +	7.829*e* − 1 (3.48*e* − 3)
	8	8.541*e* − 1 (1.27*e* − 2) −	3.945*e* − 1 (2.67*e* − 2) −	8.643*e* − 1 (1.33*e* − 2) −	8.422*e* − 1 (2.30*e* − 2) −	*8.981e* *−* *1 (4.96e* *−* *3)*
	10	8.384*e* − 1 (2.34*e* − 2) −	4.536*e* − 1 (2.17*e* − 2) −	7.993*e* − 1 (2.28*e* − 2) −	8.799*e* − 1 (1.83*e* − 2) −	*9.071e* *−* *1 (1.22e* *−* *2)*
	15	8.428*e* − 1 (6.76*e* − 2) −	2.953*e* − 1 (2.68*e* − 2) −	7.273*e* − 1 (6.12*e* − 2) −	7.828*e* − 1 (4.00*e* − 2) −	*9.459e* *−* *1 (7.02e* *−* *3)*

DTLZ3	3	3.806*e* − 1 (6.33*e* − 2) −	1.775*e* − 3 (5.02*e* − 3) −	1.001*e* − 1 (1.38*e* − 1) −	4.173*e* − 1 (5.42*e* − 1) −	*5.212e* *−* *1 (4.53e* *−* *2)*
	5	2.780*e* − 1 (2.820*e* − 1) −	2.563*e* − 1 (2.52*e* − 1) −	6.709*e* − 1 (1.95*e* − 1) =	2.316*e* − 1 (3.55*e* − 1) −	*7.688e* *−* *1 (5.15e* *−* *2)*
	8	2.736*e* − 4 (1.50*e* − 3) −	4.563*e* − 1 (2.63*e* − 2) =	2.670*e* − 1 (3.22*e* − 1) −	4.621*e* − 1 (4.13*e* − 2) =	*5.475e* *−* *1 (3.15e* *−* *1)*
	10	3.501*e* − 2 (2.42*e* − 1) −	2.511*e* − 1 (3.44*e* − 1) −	4.073*e* − 1 (2.61*e* − 1) =	4.012*e* − 1 (7.27*e* − 1) −	*4.912e* *−* *1 (4.38e* *−* *1)*
	15	5.370*e* − 1 (6.42*e* − 1) −	2.036*e* − 1 (3.54*e* − 1) −	1.996*e* − 1 (2.01*e* − 1) −	5.722*e* − 1 (5.15*e* − 1) −	*9.879e* *−* *1 (6.17e* *−* *2)*

DTLZ4	3	3.630*e* − 1 (1.69*e* − 1) −	3.023*e* − 1 (4.80*e* − 2) −	*5.359e* *−* *1 (6.60e* *−* *2)* +	3.963*e* − 1 (1.53*e* − 1) −	4.954*e* − 1 (1.03*e* − 1)
	5	5.863*e* − 1 (1.70*e* − 1) −	3.276*e* − 1 (5.26*e* − 2) −	7.872*e* − 1 (2.44*e* − 2) +	*7.973e* *−* *1 (4.00e* *−* *2) +*	7.814*e* − 1 (8.95*e* − 3)
	8	6.231*e* − 1 (1.33*e* − 1) −	3.726*e* − 1 (5.68*e* − 2) −	9.102*e* − 1 (3.20*e* − 2) −	*9.199e* *−* *1 (1.62e* *−* *2) +*	9.113*e* − 1 (6.95*e* − 3)
	10	6.928*e* − 1 (1.79*e* − 1) −	3.958*e* − 1 (7.36*e* − 2) −	9.505*e* − 1 (8.43*e* − 3) +	*9.589e* *−* *1 (5.99e* *−* *3) +*	9.396*e* − 1 (6.00*e* − 3)
	15	6.959*e* − 1 (1.80*e* − 1) −	3.394*e* − 1 (1.02*e* − 1) −	9.769*e* − 1 (9.59*e* − 3) −	9.819*e* − 1 (5.12*e* − 3) −	*9.875e* *−* *1 (8.11e* *−* *4)*

DTLZ7	3	2.288*e* − 1 (1.06*e* − 2) −	2.398*e* − 1 (1.27*e* − 2) −	*2.620e* *−* *1 (1.66e* *−* *2) +*	2.357*e* − 1 (2.32*e* − 2) −	2.526*e* − 1 (6.40*e* − 3)
	5	1.993*e* − 2 (2.06*e* − 2) −	1.805*e* − 1 (1.10*e* − 2) +	*2.069e* *−* *1 (1.34e* *−* *2)* +	1.694*e* − 1 (2.42*e* − 2) +	1.320*e* − 1 (2.08*e* − 2)
	8	2.283*e* − 3 (6.87*e* − 3) −	*1.044e* *−* *1 (1.27e* *−* *2)* +	9.639*e* − 2 (2.69*e* − 2) =	5.824*e* − 2 (3.18*e* − 2) −	9.463*e* − 2 (1.67*e* − 2)
	10	8.257*e* − 5 (1.41*e* − 4) −	1.194*e* − 1 (1.63*e* − 2) +	*1.503e* *−* *1 (3.59e* *−* *3)* +	6.109*e* − 4 (2.42*e* − 3) −	8.100*e* − 2 (1.87*e* − 3)
	15	8.583*e* − 6 (2.73*e* − 5) −	8.524*e* − 2 (2.63*e* − 2) +	*1.159e* *−* *1 (3.30e* *−* *4)* +	4.012*e* − 4 (5.11*e* − 3) −	5.400*e* − 2 (3.06*e* − 4)

*w*/*l*/*t*		0/24/1	4/20/1	8/12/5	5/18/2	

**Table 2:** The features of the test problems.

Problem	Features
DTLZ1	Linear, multimodal
DTLZ2	Concave
DTLZ3	Concave, multimodal
DTLZ4	Concave, biased
DTZL7	Mixed, disconnected, multimodal
WFG1	Mixed, biased
WFG2	Convex, disconnected, multimodal, nonseparable
WFG3	Linear, degenerate, nonseparable
WFG4	Concave, multimodal
WFG5	Concave, deceptive
WFG6	Concave, nonseparable
WFG7	Concave, biased
WFG8	Concave, biased, nonseparable
WFG9	Concave, biased, multimodal, deceptive, nonseparable

**Table 3:** The setting of the population size.

Number of objectives (*m*)	Divisions (*H*_1_, *H*_2_)	Population size (*N*)
3	(12, 0)	91
5	(6, 0)	210
8	(3, 2)	156
10	(3, 2)	275
15	(2, 1)	135

**Table 4:** The statistical results (mean and standard deviation) of the IGD values obtained by MOEA/D, dMOPSO, MOBI2, *ϵ*-MOEA, and OBEA on DTLZ1 to DTLZ4 and DTLZ7. The best results are italicized.

Problem	M	MOEAD	dMOPSO	MOMBI2	*ϵ*-MOEA	OBEA
DTLZ1	3	2.569*e* − 1 (2.50*e* − 1) =	8.076*e* + 0 (5.99*e* + 0) −	1.421*e* − 1 (1.63*e* − 1) −	3.775*e* − 1 (4.66*e* − 1) −	*1.121e* *−* *2 (5.13e* *−* *4)*
	5	2.884*e* − 1 (2.88*e* − 1) −	1.129*e* + 1 (4.53*e* + 0) −	6.283*e* − 1 (3.21*e* − 1) −	8.831*e* − 1 (5.86*e* − 1) −	*5.101e* *−* *2 (4.27e* *−* *4)*
	8	1.937*e* − 1 (9.66*e* − 2) −	5.033*e* + 0 (3.95*e* + 0) −	4.950*e* − 1 (2.04*e* − 1) −	5.220*e* + 0 (2.22*e* + 0) −	*8.202e* *−* *2 (2.51e* *−* *3)*
	10	3.256*e* − 1 (3.79*e* − 1) −	7.812*e* + 0 (3.13*e* + 0) −	5.716*e* + 0 (2.09*e* + 0) −	9.107*e* − 1 (5.31*e* − 1) −	*1.312e* *−* *1 (3.09e* *−* *3)*
	15	2.433*e* − 1 (1.20*e* − 1) =	7.121*e* + 0 (6.43*e* + 0) −	7.148*e* − 1 (5.39*e* − 1) −	6.084*e* + 0 (2.37*e* + 0) −	*1.214e* *−* *1 (3.01e* *−* *3)*

DTLZ2	3	5.492*e* − 2 (2.34*e* − 4) −	1.425*e* − 1 (8.05*e* − 3) −	5.819*e* − 2 (1.15*e* − 3) −	7.177*e* − 2 (2.78*e* − 3) −	*5.479e* *−* *2 (9.14e* *−* *5)*
	5	1.668*e* − 1 (1.91*e* − 3) =	2.619*e* − 1 (9.02*e* − 3) −	2.094*e* − 1 (4.86*e* − 3) −	*1.289e* *−* *1 (4.58e* *−* *3) +*	1.696*e* − 1 (9.83*e* − 4)
	8	3.260*e* − 1 (7.38*e* − 3) +	5.501*e* − 1 (1.88*e* − 2) −	4.079*e* − 1 (7.15*e* − 3) −	*3.102e − 1 (1.52e − 2) +*	3.335*e* − 1 (2.51*e* − 3)
	10	*4.185e* *−* *1 (2.24e* *−* *2)* *=*	6.212*e* − 1 (2.90*e* − 2) −	4.373*e* − 1 (1.67*e* − 2) −	5.073*e* − 1 (1.40*e* − 2) −	4.215*e* − 1 (3.95*e* − 3)
	15	7.053*e* − 1 (6.39*e* − 2) −	9.488*e* − 1 (3.17*e* − 2) −	8.965*e* − 1 (4.96*e* − 2) −	6.734*e* − 1 (3.81*e* − 2) −	*5.885e* *−* *1 (5.97e* *−* *3)*

DTLZ3	3	1.388*e* + 1 (9.64*e* + 0) −	4.493*e* + 1 (3.14*e* + 1) −	6.321*e* + 0 (5.45*e* + 0) −	1.719*e* + 1 (7.00*e* + 0) −	*5.494e* *−* *1 (3.21e* *−* *2)*
	5	1.933*e* + 1 (1.38*e* + 1) −	1.898*e* + 2 (3.36*e* + 1) −	1.704*e* + 1 (5.60*e* + 0) −	2.985*e* + 1 (1.07*e* + 1) −	*1.782e* *−* *1 (2.51e* *−* *3)*
	8	6.610*e* + 0 (4.88*e* + 0) −	2.032*e* + 2 (9.75*e* + 0) −	1.459*e* + 1 (5.64*e* + 0) −	1.310*e* + 2 (2.52*e* + 1) −	*8.911e* *−* *1 (8.01e* *−* *1)*
	10	6.232*e* + 0 (2.12*e* + 0) =	2.031*e* + 2 (1.22*e* + 1) −	1.530*e* + 2 (3.41*e* + 1) −	3.215*e* + 1 (7.78*e* + 0) −	*1.197e + 0 (4.32e* *−* *1)*
	15	6.469*e* + 0 (3.99*e* + 0) =	2.045*e* + 2 (7.22*e* + 0) −	1.794*e* + 1 (9.92*e* + 0) −	1.515*e* + 2 (3.44*e* + 1) −	*1.569e* *+* *0 (4.91e* *−* *1)*

DTLZ4	3	4.602*e* − 1 (3.30*e* − 1) −	3.173*e* − 1 (2.84*e* − 2) −	*1.085e* *−* *1 (1.47e* *−* *1) +*	3.695*e* − 1 (3.02*e* − 1) −	1.851*e* − 1 (2.20*e* − 1)
	5	5.470*e* − 1 (2.31*e* − 1) −	5.217*e* − 1 (2.20*e* − 2) −	2.246*e* − 1 (5.14*e* − 2) −	1.908*e* − 1 (9.86*e* − 2) −	*1.770e* *−* *1 (1.44e* *−* *2)*
	8	7.792*e* − 1 (1.35*e* − 1) −	6.505*e* − 1 (2.01*e* − 2) −	4.058*e* − 1 (6.38*e* − 2) −	*3.423e* *−* *1 (5.58e* *−* *2)* *=*	3.478*e* − 1 (1.95*e* − 3)
	10	8.279*e* − 1 (1.38*e* − 1) −	7.178*e* − 1 (1.26*e* − 2) −	*4.213e* *−* *1 (2.13e* *−* *2) +*	4.789*e* − 1 (1.80*e* − 2) −	4.631*e* − 1 (3.80*e* − 3)
	15	9.284*e* − 1 (1.14*e* − 1) −	8.328*e* − 1 (2.13*e* − 2) −	6.675*e* − 1 (2.36*e* − 2) −	*5.324e* *−* *1 (1.82e* *−* *2) +*	6.306*e* − 1 (1.38*e* − 3)

DTLZ7	3	1.907*e* − 1 (1.67*e* − 1) −	1.705*e* − 1 (1.43*e* − 1) −	1.840*e* − 1 (1.25*e* − 1) −	2.591*e* − 1 (2.71*e* − 1) =	*5.452e* *−* *2 (3.12e* *−* *3)*
	5	1.169*e* + 0 (2.71*e* − 1) −	6.097*e* − 1 (2.00*e* − 1) −	4.823*e* − 1 (1.64*e* − 1) =	6.667*e* − 1 (2.53*e* − 1) −	*4.141e* *−* *1 (5.13e* *−* *2)*
	8	1.659*e* + 0 (2.34*e* − 1) =	1.385*e* + 0 (2.73*e* − 1) =	4.324*e* + 0 (1.23*e* + 0) −	2.151*e* + 0 (6.38*e* − 1) −	*9.146e* *−* *1 (3.31e* *−* *2)*
	10	2.704*e* + 0 (5.03*e* − 1) =	2.252*e* + 0 (4.09*e* − 1) =	1.877*e* + 1 (2.27*e* + 0) −	1.007*e* + 1 (1.48*e* + 0) =	*1.452e* *+* *0 (6.76e* *−* *1)*
	15	3.181*e* + 0 (6.21*e* − 1) =	5.635*e* + 0 (1.69*e* + 0) =	2.844*e* + 1 (4.67*e* + 0) −	3.766*e* + 1 (3.27*e* + 0) −	*1.953e* *+* *0 (3.87e* *−* *1)*

*w*/*l*/*t*		1/15/9	0/22/3	2/22/1	3/19/3	

**Table 5:** The statistical results (mean and standard deviation) of the IGD values obtained by MOEA/D, dMOPSO, MOBI2, *ϵ*-MOEA, and OBEA on WFG test suits. The best results are italicized.

Problem	M	MOEAD	dMOPSO	MOMBI2	*ɛ*-MOEA	OBEA
WFG1	3	1.299*e* + 0 (8.45*e* − 2) =	1.552*e* + 0 (7.51*e* − 3) −	1.094*e* + 0 (7.15*e* − 2) −	1.517*e* + 0 (6.32*e* − 2) −	*5.083e* *−* *1 (7.94e* *−* *2)*
	5	1.998*e* + 0 (9.68*e* − 2) −	2.210*e* + 0 (3.04*e* − 2) −	1.940*e* + 0 (9.49*e* − 2) −	2.038*e* + 0 (1.74*e* − 2) −	*9.814e* *−* *1 (1.56e* *−* *1)*
	8	2.840*e* + 0 (1.72*e* − 1) −	3.125*e* + 0 (5.07*e* − 2) −	2.635*e* + 0 (2.10*e* − 1) −	2.693*e* + 0 (3.05*e* − 2) −	*1.959e* *+* *0 (3.48e* *−* *1)*
	10	3.344*e* + 0 (9.03*e* − 2) −	3.566*e* + 0 (5.15*e* − 2) −	3.070*e* + 0 (2.26*e* − 2) −	3.051*e* + 0 (1.86*e* − 1) −	*2.088e* *+* *0 (4.77e* *−* *2)*
	15	4.294*e* + 0 (8.37*e* − 2) −	4.587*e* + 0 (5.58*e* − 2) −	3.136*e* + 0 (3.54*e* − 1) =	3.972*e* + 0 (6.22*e* − 2) −	*3.115e + 0 (2.48e − 1)*

WFG2	3	1.081*e* + 0 (8.61*e* − 2) −	9.277*e* − 1 (1.79*e* − 1) −	3.477*e* − 1 (1.76*e* − 2) −	2.916*e* − 1 (5.33*e* − 2) =	*2.747e − 1 (6.59e − 2)*
	5	5.835*e* + 0 (2.62*e* − 1) −	3.811*e* + 0 (4.64*e* − 1) −	1.348*e* + 0 (1.68*e* − 1) =	*8.280e* *−* *1 (2.05e* *−* *1) +*	1.502*e* + 0 (5.72*e* − 1)
	8	8.889*e* + 0 (6.72*e* − 1) −	6.697*e* + 0 (4.23*e* − 1) −	2.169*e* + 0 (4.20*e* − 1) +	*1.482e* *+* *0 (2.97e − 1) +*	3.321*e* + 0 (8.42*e* − 1)
	10	1.696*e* + 1 (2.00*e* − 1) −	1.309*e* + 1 (2.39*e* − 1) −	*3.028e* *+* *0 (4.88e* *−* *1) +*	4.441*e* + 0 (1.41*e* + 0) =	4.661*e* + 0 (1.41*e* + 0)
	15	2.766*e* + 1 (1.71*e* − 1) −	2.274*e* + 1 (1.33*e* + 0) −	1.142*e* + 1 (2.31*e* + 0) +	*3.401e* *+* *0 (6.14e* *−* *1) +*	1.632*e* + 1 (3.19*e* + 0)

WFG3	3	4.820*e* − 1 (1.29*e* − 1) −	4.060*e* − 1 (7.70*e* − 2) −	*1.453e* *−* *1 (1.76e* *−* *2) +*	3.051*e* − 1 (3.07*e* − 2) −	1.730*e* − 1 (1.72*e* − 2)
	5	1.468*e* + 0 (3.18*e* − 1) −	7.413*e* − 1 (4.80*e* − 2) −	9.781*e* − 1 (7.32*e* − 2) −	7.482*e* − 1 (3.16*e* − 2) −	*5.355e* *−* *1 (7.74e* *−* *2)*
	8	4.279*e* + 0 (2.87*e* − 1) −	2.304*e* + 0 (5.29*e* − 1) −	3.362*e* + 0 (4.40*e* − 1) −	*1.107e* *+* *0 (3.13e* *−* *2)* +	1.208*e* + 0 (2.60*e* − 1)
	10	7.859*e* + 0 (1.16*e* + 0) −	3.300*e* + 0 (8.09*e* − 1) −	*1.263e* *+* *0 (5.16e* *−* *2) +*	3.607*e* + 0 (5.57*e* − 1) −	1.464*e* + 0 (2.81*e* − 1)
	15	1.337*e* + 1 (1.89*e* + 0) −	8.238*e* + 0 (2.91*e* + 0) −	1.035*e* + 1 (1.37*e* + 0) −	*1.493e* *+* *0 (8.05e* *−* *2)* +	2.640*e* + 0 (4.70*e* − 1)

WFG4	3	3.073*e* − 1 (1.60*e* − 2) −	4.085*e* − 1 (2.38*e* − 2) −	2.616*e* − 1 (4.73*e* − 3) −	*1.967e* *−* *1 (6.27e* *−* *3)* +	2.407*e* − 1 (3.55*e* − 3)
	5	2.095*e* + 0 (1.43*e* − 1) −	1.648*e* + 0 (1.15*e* − 1) −	1.851*e* + 0 (1.09*e* − 1) −	*8.292e* *−* *1 (5.25e* *−* *2)* +	9.865*e* − 1 (5.77*e* − 3)
	8	7.380*e* + 0 (1.28*e* − 1) −	7.561*e* + 0 (3.05*e* − 1) −	4.017*e* + 0 (5.12*e* − 1) −	3.549*e* + 0 (2.22*e* − 1) −	*2.985e* *+* *0 (1.58e* *−* *2)*
	10	9.817*e* + 0 (2.22*e* − 1) −	1.030*e* + 1 (2.35*e* − 1) −	5.938*e* + 0 (3.00*e* − 1) −	5.897*e* + 0 (3.90*e* − 1) −	*4.300e* *+* *0 (5.79e* *−* *2)*
	15	1.713*e* + 1 (3.18*e* − 1) −	1.672*e* + 1 (1.74*e* − 1) −	2.061*e* + 1 (1.20*e* + 0) −	1.316*e* + 1 (4.78*e* − 1) −	*8.888e* *+* *0 (1.17e* *−* *1)*

WFG5	3	3.099*e* − 1 (1.66*e* − 2) −	4.132*e* − 1 (3.00*e* − 2) −	2.748*e* − 1 (3.00*e* − 3) −	*2.138e* *−* *1 (9.41e* *−* *3)* +	2.463*e* − 1 (2.35*e* − 3)
	5	2.166*e* + 0 (8.60*e* − 2) −	1.314*e* + 0 (2.07*e* − 2) −	2.062*e* + 0 (7.95*e* − 2) −	*9.772e* *−* *1 (2.80e* *−* *2)* =	9.842*e* − 1 (8.25*e* − 3)
	8	7.144*e* + 0 (7.49*e* − 2) −	4.488*e* + 0 (2.12*e* − 1) −	3.767*e* + 0 (3.57*e* − 2) −	3.139*e* + 0 (1.64*e* − 1) −	*2.999e* *+* *0 (1.74e* *−* *2)*
	10	9.567*e* + 0 (1.30*e* − 1) −	5.826*e* + 0 (2.32*e* − 1) −	4.820*e* + 0 (2.18*e* − 1) −	5.511*e* + 0 (1.82*e* − 1) −	*4.266e* *+* *0 (4.17e* *−* *2)*
	15	1.631*e* + 1 (1.30*e* − 1) −	1.084*e* + 1 (3.02*e* − 1) −	2.156*e* + 1 (1.93*e* + 0) −	1.066*e* + 1 (6.76*e* − 1) −	*5.491e* *+* *0 (5.43e* *−* *2)*

WFG6	3	3.453*e* − 1 (2.07*e* − 2) −	4.387*e* − 1 (5.19*e* − 2) −	2.842*e* − 1 (7.16*e* − 3) −	2.806*e* − 1 (1.48*e* − 2) −	*2.729e* *−* *1 (8.43e* *−* *3)*
	5	2.850*e* + 0 (3.33*e* − 1) −	2.372*e* + 0 (1.64*e* − 1) −	1.972*e* + 0 (1.01*e* − 1) −	1.125*e* + 0 (2.99*e* − 2) −	*1.047e* *+* *0 (1.15e* *−* *2)*
	8	7.849*e* + 0 (1.55*e* − 1) −	8.386*e* + 0 (3.11*e* − 1) −	3.760*e* + 0 (8.83*e* − 2) −	3.268*e* + 0 (1.34*e* − 1) −	*3.044e* *+* *0 (1.51e* *−* *2)*
	10	1.005*e* + 1 (2.43*e* − 1) −	1.049*e* + 1 (2.93*e* − 1) −	4.999*e* + 0 (2.82*e* − 1) −	5.465*e* + 0 (9.81*e* − 2) −	*4.340e* *+* *0 (4.32e* *−* *2)*
	15	1.672*e* + 1 (1.88*e* − 1) −	1.686*e* + 1 (1.64*e* − 1) −	1.929*e* + 1 (1.73*e* + 0) −	1.108*e* + 1 (6.65*e* − 1) −	*5.619e* *+* *0 (4.12e* *−* *1)*

WFG7	3	4.836*e* − 1 (3.53*e* − 2) −	4.494*e* − 1 (1.34*e* − 2) −	2.607*e* − 1 (3.93*e* − 3) −	2.035*e* − 1 (1.26*e* − 2) −	*1.235e* *−* *1 (3.12e* *−* *4)*
	5	2.716*e* + 0 (3.13*e* − 1) −	1.523*e* + 0 (1.36*e* − 1) −	2.024*e* + 0 (1.06*e* − 1) −	9.271*e* − 1 (3.95*e* − 2) −	*9.132e* *−* *1 (7.13e* *−* *3)*
	8	7.776*e* + 0 (1.67*e* − 1) −	6.017*e* + 0 (1.26*e* + 0) −	3.846*e* + 0 (3.80*e* − 1) −	3.218*e* + 0 (2.54*e* − 1) −	*2.411e* *+* *0 (2.95e* *−* *2)*
	10	1.005*e* + 1 (1.88*e* − 1) −	5.473*e* + 0 (4.05*e* − 1) −	5.523*e* − 1 (1.38*e* − 2) =	7.827*e* − 1 (2.40*e* − 2) =	*5.117e* *−* *1 (1.60e* *−* *2)*
	15	1.735*e* + 1 (1.36*e* − 1) −	1.394*e* + 1 (1.98*e* + 0) −	1.735*e* + 1 (2.38*e* + 0) −	1.203*e* + 1 (7.03*e* − 1) −	*5.814e* *+* *0 (5.17e* *−* *1)*

WFG8	3	3.797*e* − 1 (2.78*e* − 2) −	5.852*e* − 1 (3.90*e* − 2) −	3.184*e* − 1 (7.85*e* − 3) −	2.9223*e* − 1 (7.87*e* − 3) =	*2.913e* *−* *1 (7.74e* *−* *3)*
	5	2.131*e* + 0 (2.08*e* − 1) −	1.538*e* + 0 (4.79*e* − 2) −	2.246*e* + 0 (6.38*e* − 2) −	1.116*e* + 0 (3.62*e* − 2) −	*1.049e* *+* *0 (6.13e* *−* *3)*
	8	6.814*e* + 0 (1.59*e* − 1) −	6.756*e* + 0 (4.51*e* − 1) −	3.973*e* + 0 (2.38*e* − 1) −	3.523*e* + 0 (1.35*e* − 1) −	*3.181e* *+* *0 (1.91e* *−* *2)*
	10	9.115*e* + 0 (2.44*e* − 1) −	8.869*e* + 0 (4.10*e* − 1) −	5.228*e* + 0 (2.71*e* − 1) −	5.871*e* + 0 (5.23*e* − 1) −	*4.762e* *+* *0 (8.61e* *−* *2)*
	15	1.324*e* + 1 (2.71*e* + 0) −	1.545*e* + 1 (3.51*e* − 1) −	1.970*e* + 1 (1.40*e* + 0) −	1.138*e* + 1 (5.51*e* − 1) −	*9.367e* *+* *0 (2.90e* *−* *1)*

WFG9	3	4.680*e* − 1 (6.68*e* − 2) −	3.495*e* − 1 (1.33*e* − 2) −	2.710*e* − 1 (1.06*e* − 2) =	2.060*e* − 1 (3.04*e* − 2) +	*1.321e* *−* *1 (5.13e* *−* *3)*
	5	2.141*e* + 0 (1.10*e* − 1) −	2.030*e* + 0 (8.89*e* − 2) −	1.954*e* + 0 (9.93*e* − 2) −	1.015*e* + 0 (4.68*e* − 2) −	*9.316e* *−* *1 (5.66e* *−* *3)*
	8	7.046*e* + 0 (2.36*e* − 1) −	6.721*e* + 0 (4.80*e* − 1) −	3.715*e* + 0 (4.70*e* − 2) −	3.276*e* + 0 (1.53*e* − 1) −	*3.104e* *+* *0 (4.92e* *−* *2)*
	10	9.240*e* + 0 (6.11*e* − 1) −	8.124*e* + 0 (7.77*e* − 1) −	5.123*e* + 0 (1.95*e* − 1) −	5.399*e* + 0 (7.48*e* − 2) −	*4.231e* *+* *0 (7.16e* *−* *2)*
	15	1.436*e* + 1 (2.01*e* + 0) −	1.132*e* + 1 (5.41*e* − 1) −	2.143*e* + 1 (1.63*e* + 0) −	1.127*e* + 1 (6.58*e* − 1) −	*5.793e* *+* *0 (6.84e* *−* *1)*

*w*/*l*/*t*		0/44/1	0/45/0	5/36/4	9/31/5	

**Table 6:** The statistical results (mean and standard deviation) of the HV values obtained by each algorithm on WFG test suits. The best results are italicized.

Problem	M	MOEAD	dMOPSO	MOMBI2	*ɛ*-MOEA	OBEA
WFG1	3	3.380*e* − 1 (3.00*e* − 2) −	2.833*e* − 1 (4.99*e* − 3) −	4.312*e* − 1 (2.66*e* − 2) −	3.009*e* − 1 (1.92*e* − 2) −	*7.613e − 1 (3.46e − 2)*
	5	3.126*e* − 1 (2.03*e* − 2) −	2.247*e* − 1 (1.19*e* − 2) −	3.514*e* − 1 (2.35*e* − 2) −	2.940*e* − 1 (2.49*e* − 3) −	*7.811e − 1 (6.25e − 1)*
	8	3.256*e* − 1 (4.42*e* − 2) =	2.088*e* − 1 (5.86*e* − 3) −	3.482*e* − 1 (6.54*e* − 2) =	2.500*e* − 1 (2.54*e* − 3) −	*4.913e − 1 (1.18e − 1)*
	10	2.799*e* − 1 (3.07*e* − 2) −	1.957*e* − 1 (8.73*e* − 3) −	2.273*e* − 1 (1.92*e* − 3) −	2.774*e* − 1 (2.62*e* − 2) −	*5.612e − 1 (4.13e − 2)*
	15	2.222*e* − 1 (1.94*e* − 2) −	1.739*e* − 1 (4.87*e* − 3) −	5.879*e* − 1 (9.09*e* − 2) −	1.903*e* − 1 (1.39*e* − 3) −	*9.913e − 1 (3.14e − 4)*

WFG2	3	7.545*e* − 1 (3.86*e* − 2) −	7.539*e* − 1 (1.35*e* − 2) −	8.653*e* − 1 (1.20*e* − 2) −	8.672*e* − 1 (2.67*e* − 2) −	*8.837e − 1 (6.87e − 3)*
	5	7.459*e* − 1 (4.20*e* − 2) −	7.732*e* − 1 (1.71*e* − 2) −	*9.332e* *−* *1 (1.13e* *−* *2)* +	8.520*e* − 1 (1.62*e* − 2) −	8.870*e* − 1 (1.17*e* − 2)
	8	7.034*e* − 1 (5.45*e* − 2) −	6.770*e* − 1 (3.34*e* − 2) −	*9.312e* *−* *1 (3.45e* *−* *2)* +	7.917*e* − 1 (2.16*e* − 2) −	9.106*e* − 1 (1.58*e* − 2)
	10	7.050*e* − 1 (5.43*e* − 2) −	6.484*e* − 1 (2.65*e* − 2) −	7.580*e* − 1 (1.78*e* − 2) −	*9.100e* *−* *1 (5.63e* *−* *2)* =	9.086*e* − 1 (1.56*e* − 2)
	15	7.480*e* − 1 (9.20*e* − 2) −	5.836*e* − 1 (5.33*e* − 2) −	7.096*e* − 1 (1.05*e* − 1) −	7.998*e* − 1 (2.76*e* − 2) −	*9.543e* *−* *1 (1.07e* *−* *2)*

WFG3	3	2.189*e* − 1 (3.28*e* − 2) −	2.512*e* − 1 (1.20*e* − 2) −	*3.648e − 1 (8.42e − 3)* +	2.672*e* − 1 (1.62*e* − 2) −	3.475*e* − 1 (8.44*e* − 3)
	5	1.511*e* − 2 (4.62*e* − 2) −	3.024*e* − 2 (1.68*e* − 2) −	4.616*e* − 2 (1.10*e* − 2) −	2.112*e* − 4 (8.31*e* − 4) −	*1.769e* *−* *1(1.28e* *−* *3)*
	8	1.201*e* − 2 (6.64*e* − 3) −	1.101*e* − 1 (6.61*e* − 2) =	2.821*e* − 3 (8.30*e* − 3) −	1.341*e* − 2 (6.12*e* − 3) =	*1.131e* *−* *1 (2.43e* *−* *3)*
	10	3.323*e* − 1 (5.13*e* − 2) −	1.012*e* − 1 (2.71*e* − 2) −	*7.487e − 1 (2.71e − 2)* *=*	5.451*e* − 1 (6.35*e* − 2) −	6.945*e* − 1 (4.55*e* − 2)
	15	2.013*e* − 1 (5.61*e* − 2) =	2.353*e* − 1 (6.89*e* − 2) =	3.267*e* − 1 (3.82*e* − 2) =	3.312*e* − 1 (5.61*e* − 2) =	*3.455e* *−* *1 (4.69e* *−* *2)*

WFG4	3	4.570*e* − 1 (1.28*e* − 2) −	4.055*e* − 1 (1.01*e* − 2) −	*5.147e − 1 (5.10e − 3)* *=*	5.103*e* − 1 (3.38*e* − 3) =	5.107*e* − 1 (2.91*e* − 3)
	5	4.955*e* − 1 (3.67*e* − 2) −	4.923*e* − 1 (2.23*e* − 2) −	5.887*e* − 1 (3.28*e* − 2) −	6.235*e* − 1 (1.16*e* − 2) −	*6.785e* *−* *1 (5.15e* *−* *3)*
	8	2.983*e* − 1 (3.58*e* − 2) −	2.195*e* − 1 (3.11*e* − 2) −	7.457*e* − 1 (5.72*e* − 2) −	6.187*e* − 1 (1.72*e* − 2) −	*7.707e* *−* *1 (7.74e* *−* *3)*
	10	2.696*e* − 1 (4.86*e* − 2) −	1.882*e* − 1 (3.08*e* − 2) −	6.134*e* − 1 (1.68*e* − 2) −	7.440*e* − 1 (3.08*e* − 2) −	*7.690e* *−* *1 (1.19e* *−* *2)*
	15	1.873*e* − 1 (6.01*e* − 2) −	6.377*e* − 2 (4.26*e* − 2) −	4.349*e* − 1 (6.33*e* − 2) −	6.256*e* − 1 (1.15*e* − 2) −	*8.486e* *−* *1 (1.13e* *−* *2)*

WFG5	3	4.399*e* − 1 (1.37*e* − 2) −	3.937*e* − 1 (1.60*e* − 2) −	4.785*e* − 1 (5.36*e* − 3) −	4.893*e* − 1 (3.76*e* − 3) −	*4.939e − 1 (4.50e − 3)*
	5	4.896*e* − 1 (3.04*e* − 2) −	4.669*e* − 1 (1.35*e* − 2) −	4.992*e* − 1 (2.96*e* − 2) −	5.023*e* − 1 (9.26*e* − 3) −	*6.303e − 1 (9.01e − 3)*
	8	3.250*e* − 1 (4.38*e* − 2) −	2.058*e* − 1 (6.14*e* − 2) −	*7.237e − 1 (1.47e − 2)* *=*	5.012*e* − 1 (1.48*e* − 2) −	7.098*e* − 1 (1.44*e* − 2)
	10	2.881*e* − 1 (4.47*e* − 2) −	1.902*e* − 1 (7.04*e* − 2) −	4.987*e* − 1 (1.31*e* − 2) −	*7.131e − 1 (2.16e − 2)* +	6.377*e* − 1 (1.79*e* − 2)
	15	1.560*e* − 1 (5.02*e* − 2) −	7.941*e* − 2 (7.57*e* − 2) −	3.269*e* − 1 (6.71*e* − 2) −	5.050*e* − 1 (1.76*e* − 2) −	*7.498e − 1 (2.03e − 2)*

WFG6	3	4.292*e* − 1 (1.30*e* − 2) −	4.035*e* − 1 (3.33*e* − 2) −	4.805*e* − 1 (7.82*e* − 3) −	4.625*e* − 1 (7.54*e* − 3) −	*5.160e − 1 (1.14e − 2)*
	5	3.586*e* − 1 (6.06*e* − 2) −	3.342*e* − 1 (2.80*e* − 2) −	5.172*e* − 1 (3.11*e* − 2) −	4.488*e* − 1 (1.01*e* − 2) −	*6.927e − 1 (1.50e − 2)*
	8	1.380*e* − 1 (3.90*e* − 2) −	1.802*e* − 1 (4.98*e* − 2) −	7.156*e* − 1 (1.66*e* − 2) =	4.533*e* − 1 (1.18*e* − 2) −	*7.598e − 1 (1.81e − 2)*
	10	1.572*e* − 1 (7.08*e* − 2) −	1.697*e* − 1 (4.19*e* − 2) −	4.502*e* − 1 (1.34*e* − 2) −	6.951*e* − 1 (2.58*e* − 2) =	*7.557e − 1 (2.25e − 2)*
	15	8.926*e* − 2 (3.35*e* − 2) −	3.924*e* − 2 (3.06*e* − 2) −	4.032*e* − 1 (5.48*e* − 2) −	4.611*e* − 1 (2.03*e* − 2) −	*7.229e − 1 (3.09e − 2)*

WFG7	3	3.592*e* − 1 (2.07*e* − 2) −	3.550*e* − 1 (8.31*e* − 3) −	*5.193e − 1 (5.85e − 3)* =	5.171*e* − 1 (6.69*e* − 3) =	5.148*e* − 1 (2.33*e* − 3)
	5	4.289*e* − 1 (5.14*e* − 2) −	4.405*e* − 1 (1.61*e* − 2) −	5.614*e* − 1 (2.86*e* − 2) −	5.455*e* − 1 (1.10*e* − 2) −	*7.182e − 1 (5.40e − 3)*
	8	2.231*e* − 1 (6.34*e* − 2) −	2.951*e* − 1 (7.75*e* − 2) −	7.746*e* − 1 (4.95*e* − 2) =	5.460*e* − 1 (1.95*e* − 2) −	*8.113e − 1 (6.13e − 3)*
	10	1.791*e* − 1 (5.47*e* − 2) −	4.767*e* − 1 (3.60*e* − 2) −	5.523*e* − 1 (1.38*e* − 2) −	7.827*e* − 1 (2.40*e* − 2) =	*8.130e − 1 (4.13e − 2)*
	15	1.144*e* − 1 (1.20*e* − 2) −	1.424*e* − 1 (1.37*e* − 1) −	5.123*e* − 1 (9.04*e* − 2) −	5.605*e* − 1 (2.20*e* − 2) −	*8.167e − 1 (4.01e − 2)*

WFG8	3	3.974*e* − 1 (1.82*e* − 2) −	2.961*e* − 1 (1.34*e* − 2) −	4.507*e* − 1 (8.06*e* − 3) −	4.492*e* − 1 (4.65*e* − 3) −	*5.122e − 1 (5.15e − 2)*
	5	2.966*e* − 1 (6.96*e* − 2) −	3.578*e* − 1 (1.01*e* − 2) −	3.722*e* − 1 (1.95*e* − 2) −	4.931*e* − 1 (1.04*e* − 2) −	*6.915e − 1 (3.63e − 3)*
	8	1.558*e* − 2 (1.85*e* − 2) −	7.033*e* − 2 (2.80*e* − 2) −	5.993*e* − 1 (1.84*e* − 2) −	4.872*e* − 1 (1.54*e* − 2) −	*6.124e − 1 (6.71e − 2)*
	10	1.407*e* − 2 (2.15*e* − 2) −	5.938*e* − 2 (1.78*e* − 2) −	4.974*e* − 1 (1.14*e* − 2) −	6.578*e* − 1 (1.43*e* − 2) =	*7.184e − 1 (5.17e − 2)*
	15	2.826*e* − 1 (3.26*e* − 1) −	4.243*e* − 2 (2.09*e* − 2) −	3.664*e* − 1 (6.96*e* − 2) −	4.970*e* − 1 (2.15*e* − 2) −	*7.841e − 1 (5.17e − 2)*

WFG9	3	3.418*e* − 1 (3.85*e* − 2) −	4.255*e* − 1 (7.30*e* − 3) −	4.817*e* − 1 (1.20*e* − 2) =	4.907*e* − 1 (1.88*e* − 2) =	*5.611e − 1 (6.12e − 3)*
	5	4.198*e* − 1 (5.69*e* − 2) −	4.868*e* − 1 (1.86*e* − 2) −	4.522*e* − 1 (3.79*e* − 2) −	5.004*e* − 1 (2.00*e* − 2) −	*6.655e − 1 (4.17e − 2)*
	8	2.284*e* − 1 (8.51*e* − 2) −	2.534*e* − 1 (3.38*e* − 2) −	6.782*e* − 1 (3.63*e* − 2) =	4.898*e* − 1 (1.31*e* − 2) −	*7.012e − 1 (6.32e − 2)*
	10	1.441*e* − 1 (7.10*e* − 2) −	2.545*e* − 1 (2.71*e* − 2) −	4.898*e* − 1 (1.66*e* − 2) −	6.675*e* − 1 (3.17*e* − 2) =	*6.791e − 1 (4.17e − 2)*
	15	2.060*e* − 1 (1.80*e* − 1) −	2.494*e* − 1 (8.73*e* − 2) −	3.918*e* − 1 (7.50*e* − 2) −	5.196*e* − 1 (2.05*e* − 2) −	*7.824e − 1 (3.42e − 2)*

*w*/*l*/*t*		0/43/2	0/43/2	3/32/10	1/34/10	

**Table 7:** The statistical results (mean and standard deviation) of the IGD values obtained by RVEA, NSGA-III, SPEAR, MOEA/DD, Two_Arch2, MOEA/DVA, and OBEA on DTLZ test suits. The best results are italicized.

Problem	M	RVEA	NSGA-III	SPEAR	MOEA/DD	Two_Arch2	MOEA/DVA	OBEA
DTLZ1	3	4.997*e* − 1 (3.35*e* − 1) −	2.608*e* − 1 (2.03*e* − 1) −	1.100*e* − 1 (1.30*e* − 1) −	4.459*e* − 1 (2.52*e* − 1) −	4.650*e* − 2 (2.52*e* − 2) −	3.599*e* − 1 (2.57*e* − 1) −	*1.121e − 2 (5.13e − 4)*
	5	1.102*e* + 0 (5.51*e* − 1) −	1.511*e* + 0 (5.80*e* − 1) −	1.416*e* − 1 (7.28*e* − 2) −	1.030*e* + 0 (4.11*e* − 1) −	6.561*e* − 2 (6.73*e* − 3) −	5.957*e* − 2 (2.36*e* − 3) −	*5.101e − 2 (4.27e − 4)*
	8	6.312*e* − 1 (3.18*e* − 1) −	1.960*e* + 0 (6.81*e* − 1) −	2.331*e* − 1 (6.85*e* − 2) −	5.884*e* − 1 (2.74*e* − 1) −	1.190*e* − 1 (1.61*e* − 2) −	1.034*e* − 1 (1.24*e* − 3) −	*8.202e − 2 (2.51e − 3)*
	10	9.947*e* − 1 (3.36*e* − 1) −	3.182*e* + 0 (1.57*e* + 0) −	5.290*e* − 1 (2.83*e* − 1) −	1.057*e* + 0 (6.06*e* − 1) −	1.453*e* − 1 (2.12*e* − 2) −	*1.119e − 1 (7.27e − 4)* =	1.312*e* − 1 (3.09*e* − 3)
	15	3.456*e* − 1 (1.19*e* − 1) =	1.189*e* + 0 (5.61*e* − 1) −	7.744*e* + 0 (4.56*e* + 0) −	3.937*e* − 1 (2.88*e* − 1) =	2.518*e* − 1 (1.01*e* − 1) −	1.332*e* − 1 (1.88*e* − 3) =	*1.214e − 1 (3.01e − 3)*

DTLZ2	3	5.569*e* − 2 (4.96*e* − 4) −	5.495*e* − 2 (1.42*e* − 4) −	7.230*e* − 2 (2.57*e* − 3) −	5.541*e* − 2 (4.55*e* − 4) =	*3.644e − 2 (6.21e − 4)* +	5.164*e* − 2 (9.58*e* − 4) =	5.479*e* − 2 (9.14*e* − 5)
	5	1.759*e* − 1 (2.14*e* − 3) −	1.859*e* − 1 (3.63*e* − 3) −	2.154*e* − 1 (1.13*e* − 2) −	1.725*e* − 1 (1.76*e* − 3) =	2.106*e* − 1 (1.61*e* − 3) −	2.044*e* − 1 (2.70*e* − 3) −	*1.696e − 1 (9.83e − 4)*
	8	3.289*e* − 1 (1.99*e* − 3) +	4.330*e* − 1 (1.00*e* − 1) −	4.297*e* − 1 (4.62*e* − 2) −	*3.285e − 1 (2.60e − 3) +*	3.512*e* − 1 (2.88*e* − 3) =	3.167*e* − 1 (1.98*e* − 3) =	3.335*e* − 1 (2.51*e* − 3)
	10	4.239*e* − 1 (4.74*e* − 3) =	6.047*e* − 1 (8.69*e* − 2) −	8.016*e* − 1 (4.78*e* − 2) −	4.233*e* − 1 (5.31*e* − 3) =	4.788*e* − 1 (6.39*e* − 3) −	4.689*e* − 1 (2.62*e* − 3) −	*4.215e − 1 (3.95e − 3)*
	15	5.968*e* − 1 (1.77*e* − 2) =	7.367*e* − 1 (5.21*e* − 2) −	7.433*e* − 1 (4.78*e* − 2) −	6.068*e* − 1 (2.31*e* − 2) −	6.179*e* − 1 (6.59*e* − 3) −	*5.086e − 1 (2.58e − 3)* +	5.885*e* − 1 (5.97*e* − 3)

DTLZ3	3	1.546*e* + 1 (4.20*e* + 0) −	1.038*e* + 1 (3.51*e* + 0) −	8.519*e* − 1 (7.73*e* − 1) =	1.794*e* + 1 (5.90*e* + 0) −	2.827*e* + 0 (1.13*e* + 0) −	5.682*e* + 1 (1.18*e* + 1) −	*5.494e − 1 (3.21e − 2)*
	5	4.328*e* + 1 (1.02*e* + 1) −	5.736*e* + 1 (1.20*e* + 1) −	1.165*e* + 1 (6.04*e* + 0) −	5.004*e* + 1 (1.05*e* + 1) −	1.779*e* + 0 (1.23*e* + 0) −	4.175*e* − 1 (2.50*e* − 1) −	*1.782e − 1 (2.51e − 3)*
	8	2.744*e* + 1 (9.59*e* + 0) −	7.911*e* + 1 (2.93*e* + 1) −	4.192*e* + 0 (2.77*e* + 0) −	3.024*e* + 1 (8.72*e* + 0) −	7.705*e* + 0 (2.79*e* + 0) −	2.086*e* + 0 (1.77*e* + 0) −	*8.911e − 1 (8.01e − 1)*
	10	4.608*e* + 1 (1.38*e* + 1) −	1.403*e* + 2 (2.52*e* + 1) −	3.490*e* + 1 (1.11*e* + 1) −	5.597*e* + 0 (1.09*e* + 1) −	1.484*e* + 1 (2.95*e* + 0) −	2.567*e* + 0 (9.06*e* − 1) =	*1.197e + 0 (4.32e − 1)*
	15	1.996*e* + 1 (7.94*e* + 0) −	1.360*e* + 2 (5.17*e* + 1) −	1.968*e* + 2 (6.42*e* + 1) −	2.072*e* + 0 (9.46*e* + 0) −	4.698*e* + 1 (9.88*e* + 0) −	2.117*e* + 0 (1.20*e* + 0) −	*1.569e + 0 (4.91e − 1)*

DTLZ4	3	5.607*e* − 2 (6.32*e* − 4) +	1.686*e* − 1 (2.09*e* − 1) +	8.126*e* − 2 (1.65*e* − 2) +	5.548*e* − 2 (3.79*e* − 4) +	*3.623e − 2 (4.36e − 4)* +	5.949*e* − 1 (1.81*e* − 3) −	1.851*e* − 1 (2.20*e* − 1)
	5	1.794*e* − 1 (3.97*e* − 3) −	1.997*e* − 1 (4.50*e* − 2) −	4.009*e* − 1 (2.69*e* − 2) −	1.780*e* − 1 (4.35*e* − 3) =	1.663*e* − 1 (2.42*e* − 3) =	*1.558e − 1 (6.58e − 4)* =	1.770*e* − 1 (1.44*e* − 2)
	8	3.489*e* − 1 (1.15*e* − 2) =	4.614*e* − 1 (9.89*e* − 2) −	5.482*e* − 1 (3.18*e* − 2) −	3.539*e* − 1 (2.72*e* − 2) =	3.510*e* − 1 (3.34*e* − 3) =	*3.192e − 1 (3.70e − 4)* =	3.478*e* − 1 (1.95*e* − 3)
	10	4.640*e* − 1 (4.80*e* − 3) =	5.677*e* − 1 (4.65*e* − 2) −	6.921*e* − 1 (2.76*e* − 2) −	4.763*e* − 1 (1.28*e* − 2) =	*4.285e − 1 (3.09e − 3)* =	4.713*e* − 1 (2.57*e* − 2) −	4.631*e* − 1 (3.80*e* − 3)
	15	6.366*e* − 1 (9.63*e* − 3) =	8.185*e* − 1 (6.48*e* − 2) −	8.189*e* − 1 (4.44*e* − 2) −	6.413*e* − 1 (1.21*e* − 2) −	5.670*e* − 1 (1.11*e* − 2) =	*5.285e − 1 (1.30e − 2)* +	6.306*e* − 1 (1.38*e* − 3)

DTLZ7	3	1.934*e* − 1 (3.76*e* − 2) −	1.204*e* − 1 (7.52*e* − 2) =	2.814*e* − 1 (7.45*e* − 2) −	5.516*e* − 1 (2.48*e* − 1) =	4.901*e* − 2 (3.96*e* − 3) +	*4.045e − 2 (1.08e − 3)* +	5.452*e* − 2 (3.12*e* − 3)
	5	8.014*e* − 1 (1.00*e* − 1) −	8.126*e* − 1 (1.19*e* − 1) −	1.460*e* + 0 (4.28*e* − 1) −	*2.045e + 0 (4.82e − 1)* +	2.046*e* − 1 (9.47*e* − 3) +	3.023*e* − 1 (2.62*e* − 2) +	4.141*e* − 1 (5.13*e* − 2)
	8	1.621*e* + 0 (6.52*e* − 1) =	4.793*e* + 0 (7.44*e* − 1) −	5.423*e* + 0 (1.09*e* + 0) −	1.574*e* + 0 (4.65*e* − 1) −	*6.024e − 1 (1.86e − 2)* +	1.282*e* + 0 (2.53*e* − 1) −	9.146*e* − 1 (3.31*e* − 2)
	10	3.657*e* + 0 (1.87*e* + 0) =	1.325*e* + 1 (1.41*e* + 0) −	1.656*e* + 1 (2.73*e* + 0) −	1.530*e* + 0 (1.13*e* − 1) =	1.614*e* + 0 (4.31*e* − 2) =	2.094*e* + 0 (2.68*e* − 1) =	*1.452e + 0 (6.76e − 1)*
	15	5.228*e* + 0 (2.67*e* + 0) =	1.779*e* + 1 (2.38*e* + 0) −	1.896*e* + 1 (1.78*e* + 0) −	3.201*e* + 0 (1.22*e* − 1) −	1.078*e* + 1 (7.13*e* − 1) −	3.241*e* + 0 (3.75*e* − 1) −	*1.953e + 0 (3.87e − 1)*

*w*/*l*/*t*		2/14/9	1/23/1	2/22/1	3/13/9	5/14/6	4/15/6	

**Table 8:** The statistical results (mean and standard deviation) of the IGD values obtained by RVEA, NSGA-III, SPEAR, MOEA/DD, Two_Arch2, MOEA/DVA, and OBEA on WFG test suits. The best results are italicized.

Problem	M	RVEA	NSGA-III	SPEAR	MOEA/DD	Two_Arch2	MOEA/DVA	OBEA
WFG1	3	1.353*e* + 0 (5.32*e* − 2) −	1.351*e* + 0 (3.94*e* − 2) −	1.943*e* + 0 (3.57*e* − 2) −	1.505*e* + 0 (5.41*e* − 2) −	1.158*e* + 0 (4.94*e* − 2) −	1.358*e* + 0 (2.63*e* − 2) −	*5.083e − 1 (7.94e − 2)*
	5	1.924*e* + 0 (3.36*e* − 2) −	1.992*e* + 0 (3.41*e* − 2) −	2.387*e* + 0 (4.86*e* − 2) −	2.201*e* + 0 (1.03*e* − 1) −	1.627*e* + 0 (7.58*e* − 2) −	1.733*e* + 0 (3.00*e* − 2) −	*9.814e − 1 (1.56e − 1)*
	8	2.564*e* + 0 (5.35*e* − 2) −	2.686*e* + 0 (4.65*e* − 2) =	3.072*e* + 0 (3.53*e* − 1) −	2.790*e* + 0 (3.35*e* − 2) −	2.436*e* + 0 (5.48*e* − 2) −	2.780*e* + 0 (6.81*e* − 2) −	*1.959e + 0 (3.48e − 1)*
	10	2.924*e* + 0 (5.26*e* − 2) −	3.154*e* + 0 (5.28*e* − 2) −	3.327*e* + 0 (8.51*e* − 2) −	3.108*e* + 0 (3.10*e* − 2) =	2.799*e* + 0 (3.64*e* − 2) −	3.252*e* + 0 (5.35*e* − 2) −	*2.088e + 0 (4.77e − 2)*
	15	3.518*e* + 0 (2.28*e* − 1) =	4.022*e* + 0 (1.91*e* − 1) −	3.997*e* + 0 (2.70*e* − 1) =	4.272*e* + 0 (7.06*e* − 2) =	3.790*e* + 0 (3.90*e* − 2) −	4.230*e* + 0 (7.38*e* − 2) −	*3.115e + 0 (2.48e − 1)*

WFG2	3	3.055*e* − 1 (2.62*e* − 2) −	2.262*e* − 1 (1.78*e* − 2) −	7.566*e* − 1 (2.46*e* − 1) −	4.168*e* − 1 (1.08*e* − 1) −	*1.210e − 1 (5.90e − 3)* +	4.002*e* − 1 (2.45*e* − 2) −	2.747*e* − 1 (6.59*e* − 2)
	5	1.190*e* + 0 (4.01*e* − 1) =	6.904*e* − 1 (6.05*e* − 2) −	4.437*e* + 0 (1.64*e* + 0) −	2.753*e* + 0 (4.53*e* − 1) −	*6.203e − 1 (1.06e − 1)* +	1.372*e* + 0 (1.77*e* − 1) =	1.502*e* + 0 (5.72*e* − 1)
	8	4.110*e* + 0 (9.56*e* − 1) −	*1.084e + 0 (1.17e − 1)* +	5.338*e* + 0 (1.98*e* + 0) −	4.883*e* + 0 (8.37*e* − 1) −	1.331*e* + 0 (4.07*e* − 1) =	4.234*e* + 0 (3.15*e* − 1) −	3.321*e* + 0 (8.42*e* − 1)
	10	6.072*e* + 0 (2.26*e* + 0) =	*1.379e + 0 (1.47e − 1)* +	5.298*e* + 0 (1.63*e* + 0) =	8.855*e* + 0 (1.82*e* + 0) −	2.141*e* + 0 (9.47*e* − 2) +	8.325*e* + 0 (3.81*e* + 0) −	4.661*e* + 0 (1.41*e* + 0)
	15	1.213*e* + 1 (3.43*e* + 0) =	4.156*e* + 0 (1.46*e* + 0) −	2.602*e* + 0 (6.92*e* − 1) +	1.776*e* + 1 (2.05*e* + 0) =	*2.201e + 0 (7.24e − 1)* +	2.078*e* + 1 (4.53*e* + 0) −	1.632*e* + 1 (3.19*e* + 0)

WFG3	3	3.070*e* − 1 (2.98*e* − 2) −	2.471*e* − 1 (3.06*e* − 3) −	4.032*e* − 1 (5.37*e* − 2) −	3.944*e* − 1 (9.74*e* − 2) −	1.153*e* − 1 (1.33*e* − 2) =	*8.484e − 2 (7.19e − 3)* +	1.730*e* − 1 (1.72*e* − 2)
	5	7.938*e* − 1 (1.39*e* − 1) −	1.005*e* + 0 (7.46*e* − 3) −	8.296*e* − 1 (1.26*e* − 1) −	7.444*e* − 1 (5.32*e* − 2) −	3.525*e* − 1 (1.92*e* − 2) =	1.225*e* + 0 (1.48*e* − 1) −	*5.355e − 1 (7.74e − 2)*
	8	1.895*e* + 0 (3.00*e* − 1) −	3.039*e* + 0 (8.46*e* − 2) −	5.639*e* + 0 (4.82*e* − 1) −	2.037*e* + 0 (1.45*e* − 1) =	*9.309e − 1 (5.24e − 2)* =	3.879*e* + 0 (1.91*e* − 1) −	1.208*e* + 0 (2.60*e* − 1)
	10	2.323*e* + 0 (2.08*e* − 1) −	4.409*e* + 0 (1.46*e* − 1) −	5.276*e* + 0 (1.03*e* + 0) −	2.588*e* + 0 (1.18*e* − 1) =	*1.228e + 0 (1.65e − 1)* =	5.244*e* + 0 (2.45*e* − 1) −	1.464*e* + 0 (2.81*e* − 1)
	15	5.784*e* + 0 (7.08*e* − 1) −	9.537*e* + 0 (4.60*e* − 1) −	3.226*e* + 0 (2.48*e* − 1) −	5.694*e* + 0 (2.75*e* − 1) −	2.829*e* + 0 (1.45*e* − 1) −	1.229*e* + 1 (2.95*e* + 0) −	*2.640e + 0 (4.70e − 1)*

WFG4	3	2.816*e* − 1 (8.35*e* − 3) −	2.512*e* − 1 (3.64*e* − 3) −	3.248*e* − 1 (1.22*e* − 2) −	2.478*e* − 1 (2.46*e* − 3) =	2.626*e* − 1 (3.78*e* − 3) −	*1.972e − 1 (5.39e − 3)* +	2.407*e* − 1 (3.55*e* − 3)
	5	9.940*e* − 1 (5.27*e* − 3) −	1.033*e* + 0 (9.27*e* − 3) −	1.098*e* + 0 (2.28*e* − 2) −	1.057*e* + 0 (5.30*e* − 3) −	*9.397e − 1 (7.75e − 3)* =	1.391*e* + 0 (9.82*e* − 2) −	9.865*e* − 1 (5.77*e* − 3)
	8	3.151*e* + 0 (8.52*e* − 2) −	3.050*e* + 0 (3.27*e* − 2) −	3.219*e* + 0 (6.05*e* − 2) −	3.507*e* + 0 (1.71*e* − 1) −	*2.938e + 0 (9.92e − 3)* =	4.110*e* + 0 (9.22*e* − 2) −	2.985*e* + 0 (1.58*e* − 2)
	10	4.430*e* + 0 (1.82*e* − 1) −	4.368*e* + 0 (5.06*e* − 2) −	4.458*e* + 0 (1.05*e* − 1) −	4.424*e* + 0 (7.57*e* − 2) =	4.335*e* + 0 (2.19*e* − 2) =	5.886*e* + 0 (2.28*e* − 1) −	*4.300e + 0 (5.79e − 2)*
	15	9.522*e* + 0 (7.92*e* − 1) −	9.014*e* + 0 (3.68*e* − 1) −	9.325*e* + 0 (2.04*e* − 1) −	9.363*e* + 0 (8.00*e* − 1) =	*8.384e + 0 (1.43e − 2)* +	1.070*e* + 1 (1.17*e* − 1) −	8.888*e* + 0 (1.17*e* − 1)

WFG5	3	2.840*e* − 1 (1.06*e* − 2) −	2.837*e* − 1 (1.27*e* − 2) −	3.226*e* − 1 (1.51*e* − 2) −	2.523*e* − 1 (3.85*e* − 3) =	1.767*e* − 1 (4.10*e* − 3) −	2.857*e* − 1 (5.13*e* − 3) =	*2.463e − 1 (2.35e − 3)*
	5	1.015*e* + 0 (8.88*e* − 3) −	1.093*e* + 0 (1.84*e* − 2) −	1.041*e* + 0 (1.75*e* − 2) −	1.051*e* + 0 (6.78*e* − 3) −	9.864*e* − 1 (5.11*e* − 3) =	1.311*e* + 0 (1.17*e* − 1) −	*9.842e − 1 (8.25e − 3)*
	8	3.257*e* + 0 (4.78*e* − 2) −	3.102*e* + 0 (3.54*e* − 2) −	3.257*e* + 0 (5.83*e* − 2) −	3.443*e* + 0 (1.15*e* − 1) −	*2.912e + 0 (2.35e − 2)* =	3.902*e* + 0 (1.61*e* − 1) =	2.999*e* + 0 (1.74*e* − 2)
	10	*4.169e + 0 (2.21e − 2)* +	4.459*e* + 0 (8.45*e* − 2) −	4.540*e* + 0 (1.23*e* − 1) −	4.472*e* + 0 (1.01*e* − 1) =	4.324*e* + 0 (1.67*e* − 2) =	6.169*e* + 0 (1.46*e* − 1) −	4.266*e* + 0 (4.17*e* − 2)
	15	8.616*e* + 0 (3.72*e* − 1) =	9.206*e* + 0 (2.96*e* − 1) −	9.056*e* + 0 (7.88*e* − 2) −	9.132*e* + 0 (6.34*e* − 1) −	8.047*e* + 0 (8.66*e* − 2) −	1.104*e* + 1 (2.19*e* − 1) −	*5.491e + 0 (5.43e − 2)*

WFG6	3	3.475*e* − 1 (1.15*e* − 2) −	2.528*e* − 1 (6.39*e* − 3) −	3.671*e* − 1 (3.40*e* − 2) −	3.273*e* − 1 (4.27*e* − 2) =	2.101*e* − 1 (1.10*e* − 2) −	*2.562e − 1 (6.03e − 3)* =	2.729*e* − 1 (8.43*e* − 3)
	5	1.074*e* + 0 (1.65*e* − 2) −	1.056*e* + 0 (1.08*e* − 2) −	1.198*e* + 0 (3.44*e* − 2) −	1.095*e* + 0 (1.09*e* − 2) =	*9.695e − 1 (1.48e − 2)* =	1.780*e* + 0 (1.40*e* − 1) =	1.047*e* + 0 (1.15*e* − 2)
	8	3.378*e* + 0 (7.79*e* − 2) −	3.058*e* + 0 (4.75*e* − 2) −	3.274*e* + 0 (6.16*e* − 2) −	3.363*e* + 0 (6.95*e* − 2) =	*2.989e + 0 (2.55e − 2)* =	4.771*e* + 0 (2.08*e* − 1) =	3.044*e* + 0 (1.51*e* − 2)
	10	*4.221e + 0 (4.75e − 2)* +	6.636*e* − 1 (2.32*e* − 2) −	4.588*e* + 0 (1.98*e* − 1) −	4.487*e* + 0 (7.94*e* − 2) =	4.406*e* + 0 (2.91*e* − 2) −	6.915*e* + 0 (5.56*e* − 2) −	4.340*e* + 0 (4.32*e* − 2)
	15	8.478*e* + 0 (4.55*e* − 1) −	1.024*e* + 1 (4.25*e* − 1) −	9.376*e* + 0 (9.01*e* − 2) −	8.911*e* + 0 (1.81*e* − 1) −	8.161*e* + 0 (9.22*e* − 2) −	1.279*e* + 1 (2.08*e* − 1) −	*5.619e + 0 (4.12e − 1)*

WFG7	3	3.382*e* − 1 (3.13*e* − 2) −	2.984*e* − 1 (8.05*e* − 3) −	3.286*e* − 1 (1.14*e* − 2) −	2.677*e* − 1 (1.59*e* − 2) −	1.524*e* − 1 (3.15*e* − 3) −	2.018*e* − 1 (7.59*e* − 3) −	*1.235e − 1 (3.12e − 4)*
	5	1.037*e* + 0 (1.43*e* − 2) −	1.089*e* + 0 (1.31*e* − 2) −	1.133*e* + 0 (2.00*e* − 2) −	1.083*e* + 0 (9.64*e* − 3) =	9.253*e* − 1 (3.99*e* − 3) =	1.771*e* + 0 (1.28*e* − 1) −	*9.132e − 1 (7.13e − 3)*
	8	3.216*e* + 0 (6.04*e* − 2) −	3.404*e* + 0 (2.32*e* − 1) −	3.245*e* + 0 (4.64*e* − 2) −	3.279*e* + 0 (5.50*e* − 2) =	2.897*e* + 0 (3.33*e* − 2) −	4.807*e* + 0 (1.21*e* − 1) −	*2.411e + 0 (2.95e − 2)*
	10	6.658*e* − 1 (2.40*e* − 2) −	4.936*e* + 0 (2.81*e* − 1) =	6.574*e* − 1 (1.76*e* − 2) −	4.403*e* + 0 (5.19*e* − 2) −	4.299*e* + 0 (1.62*e* − 2) −	6.881*e* + 0 (2.49*e* − 1) −	*5.117e − 1 (1.60e − 2)*
	15	9.214*e* + 0 (1.72*e* − 1) −	1.090*e* + 1 (7.17*e* − 1) −	9.536*e* + 0 (2.47*e* − 1) −	9.318*e* + 0 (3.92*e* − 1) −	8.304*e* + 0 (6.85*e* − 2) −	1.062*e* + 1 (1.90*e* − 1) −	*5.814e + 0 (5.17e − 1)*

WFG8	3	3.484*e* − 1 (1.09*e* − 2) −	2.850*e* − 1 (1.70*e* − 2) −	4.027*e* − 1 (1.65*e* − 2) −	3.429*e* − 1 (2.42*e* − 2) −	*2.3762e − 1 (5.96e − 3)* +	2.510*e* − 1 (6.15*e* − 3) +	2.913*e* − 1 (7.74*e* − 3)
	5	1.082*e* + 0 (8.50*e* − 3) =	1.091*e* + 0 (2.83*e* − 2) =	1.175*e* + 0 (1.30*e* − 2) =	1.154*e* + 0 (2.36*e* − 2) =	1.120*e* + 0 (9.66*e* − 1) −	1.622*e* + 0 (6.49*e* − 2) =	*1.049e + 0 (6.13e − 3)*
	8	3.264*e* + 0 (9.49*e* − 2) =	3.205*e* + 0 (7.42*e* − 2) =	3.642*e* + 0 (3.95*e* − 1) −	3.397*e* + 0 (3.91*e* − 2) =	3.205*e* + 0 (1.65*e* − 2) =	4.547*e* + 0 (2.61*e* − 1) =	*3.181e + 0 (1.91e − 2)*
	10	4.756*e* + 0 (1.55*e* − 1) =	4.611*e* + 0 (9.60*e* − 2) −	6.357*e* + 0 (3.26*e* − 1) −	*4.581e + 0 (3.99e − 2)* =	4.781*e* + 0 (4.29*e* − 2) =	6.335*e* + 0 (1.22*e* − 1) −	4.762*e* + 0 (8.61*e* − 2)
	15	9.213*e* + 0 (6.79*e* − 1) =	9.433*e* + 0 (3.07*e* − 1) −	9.600*e* + 0 (1.93*e* − 1) −	9.308*e* + 0 (3.98*e* − 1) =	*9.047e + 0 (2.10e − 1)* =	9.756*e* + 0 (2.89*e* − 1) −	9.367*e* + 0 (2.90*e* − 1)

WFG9	3	3.481*e* − 1 (3.38*e* − 2) −	2.307*e* − 1 (2.88*e* − 2) =	3.063*e* − 1 (1.02*e* − 2) −	2.651*e* − 1 (1.86*e* − 2) −	1.682*e* − 1 (2.26*e* − 2) −	2.338*e* − 1 (4.28*e* − 3) −	*1.321e − 1 (5.13e − 3)*
	5	1.073*e* + 0 (2.43*e* − 2) −	9.430*e* − 1 (2.99*e* − 1) =	1.022*e* + 0 (1.74*e* − 2) −	1.091*e* + 0 (1.99*e* − 2) −	9.323*e* − 1 (8.43*e* − 3) =	1.281*e* + 0 (3.44*e* − 2) =	*9.316e − 1 (5.66e − 3)*
	8	3.161*e* + 0 (5.55*e* − 2) =	*2.331e + 0 (1.27e + 0) +*	3.263*e* + 0 (5.85*e* − 2) =	3.362*e* + 0 (5.21*e* − 2) −	3.055*e* + 0 (8.28*e* − 2) =	3.680*e* + 0 (1.81*e* − 1) =	3.104*e* + 0 (4.92*e* − 2)
	10	4.365*e* + 0 (1.40*e* − 1) =	*3.608e + 0 (1.06e + 0) +*	4.821*e* + 0 (3.50*e* − 1) −	4.479*e* + 0 (9.37*e* − 2) =	4.413*e* + 0 (6.04*e* − 2) =	5.541*e* + 0 (2.92*e* − 2) =	4.231*e* + 0 (7.16*e* − 2)
	15	8.560*e* + 0 (2.38*e* − 1) =	1.111*e* + 1 (2.84*e* + 0) −	9.011*e* + 0 (6.74*e* − 2) −	8.953*e* + 0 (2.49*e* − 1) =	8.493*e* + 0 (1.18*e* − 1) −	9.714*e* + 0 (2.07*e* − 1) −	*5.793e + 0 (6.84e − 1)*

*w*/*l*/*t*		2/31/12	4/32/6	1/40/4	0/23/22	6/19/20	3/31/11	

**Table 9:** The statistical results (mean and standard deviation) of the HV values obtained by RVEA, NSGA-III, SPEAR, MOEA/DD, Two_Arch2, MOEA/DVA, and OBEA on DTLZ test suits. The best results are italicized.

Problem	M	RVEA	NSGA-III	SPEAR	MOEA/DD	Two_Arch2	MOEA/DVA	OBEA
DTLZ1	3	1.381*e* − 1 (2.16*e* − 1) −	3.376*e* − 1 (3.13*e* − 1) −	6.201*e* − 1 (2.52*e* − 1) =	1.410*e* − 1 (2.11*e* − 1) −	7.960*e* − 1 (5.40*e* − 2) −	2.046*e* − 1 (2.74*e* − 1) −	*8.471e − 1 (6.13e − 3)*
	5	8.322*e* − 3 (2.82*e* − 2) −	3.785*e* − 4 (2.07*e* − 3) −	7.918*e* − 1 (2.04*e* − 1) −	6.365*e* − 3 (2.85*e* − 2) −	9.706*e* − 1 (3.70*e* − 3) −	9.327*e* − 1 (1.45*e* − 2) −	*9.763e − 1 (4.15e − 4)*
	8	1.372*e* − 1 (2.09*e* − 1) −	7.559*e* − 1 (3.35*e* − 1) −	7.544*e* − 1 (1.72*e* − 1) −	1.647*e* − 1 (2.57*e* − 1) −	9.770*e* − 1 (1.16*e* − 2) =	*9.847e − 1 (3.07e − 4) =*	9.524*e* − 1 (6.84*e* − 3)
	10	2.852*e* − 3 (7.36*e* − 3) −	3.791*e* − 1 (4.86*e* − 1) −	2.673*e* − 1 (2.73*e* − 1) −	1.034*e* − 2 (3.15*e* − 2) −	9.548*e* − 1 (4.52*e* − 2) =	*9.899e − 1 (6.59e − 4) =*	9.576*e* − 1 (4.18*e* − 3)
	15	3.745*e* − 1 (3.30*e* − 1) −	2.847*e* − 2 (8.42*e* − 2) −	6.304*e* − 1 (4.22*e* − 2) −	4.646*e* − 1 (3.17*e* − 1) −	6.639*e* − 1 (3.42*e* − 1) =	9.161*e* − 1 (5.54*e* − 3) =	*9.456e − 1 (4.46e − 6)*

DTLZ2	3	5.531*e* − 1 (1.01*e* − 3) −	5.554*e* − 1 (6.39*e* − 4) −	5.349*e* − 1 (4.08*e* − 3) −	5.546*e* − 1 (9.79*e* − 4) =	*5.778e − 1 (1.97e − 4)* =	5.373*e* − 1 (7.82*e* − 4) =	5.563*e* − 1 (5.70*e* − 4)
	5	7.682*e* − 1 (6.07*e* − 3) −	7.455*e* − 1 (8.96*e* − 3) −	7.619*e* − 1 (7.89*e* − 3) −	7.693*e* − 1 (5.35*e* − 3) =	7.232*e* − 1 (7.10*e* − 3) −	7.629*e* − 1 (6.20*e* − 3) =	*7.829e − 1 (3.48e − 3)*
	8	8.938*e* − 1 (6.36*e* − 3) −	7.789*e* − 1 (7.29*e* − 2) −	8.545*e* − 1 (3.38*e* − 2) −	8.708*e* − 1 (1.08*e* − 2) =	7.844*e* − 1 (1.28*e* − 2) −	*9.204e − 1 (3.92e − 3) =*	8.981*e* − 1 (4.96*e* − 3)
	10	9.011*e* − 1 (1.16*e* − 2) =	6.419*e* − 1 (8.57*e* − 2) −	5.809*e* − 1 (7.73*e* − 2) −	8.598*e* − 1 (2.14*e* − 2) −	6.907*e* − 1 (2.68*e* − 2) −	8.197*e* − 1 (1.62*e* − 3) −	*9.071e − 1 (1.22e − 2)*
	15	*9.487e − 1 (1.24e − 2)* =	6.697*e* − 1 (9.14*e* − 2) −	4.951*e* − 1 (1.43*e* − 1) −	9.425*e* − 1 (1.25*e* − 2) =	5.704*e* − 1 (1.81*e* − 2) −	9.060*e* − 1 (3.08*e* − 3) =	9.459*e* − 1 (7.02*e* − 3)

DTLZ3	3	2.2193*e* − 1 (2.34*e* − 1) −	2.819*e* − 1 (1.32*e* − 1) −	1.466*e* − 1 (4.62*e* − 1) −	*6.061e − 1 (1.64e − 2) +*	2.475*e* − 1 (2.63*e* − 1) −	2.032*e* − 1 (6.63*e* − 1) −	5.212*e* − 1 (4.53*e* − 2)
	5	4.536*e* − 1 (4.22*e* − 1) −	5.753*e* − 1 (2.35*e* − 1) −	3.244*e* − 1 (2.25*e* − 1) −	5.042*e* − 1 (1.30*e* − 2) −	6.200*e* − 2 (1.29*e* − 1) −	4.499*e* − 1 (2.67*e* − 1) −	*7.688e − 1 (5.15e − 2)*
	8	3.255*e* − 1 (6.33*e* − 2) −	4.822*e* − 1 (3.56*e* − 1) =	4.631*e* − 1 (3.12*e* − 2) =	4.152*e* − 1 (1.80*e* − 2) −	3.344*e* − 1 (3.55*e* − 1) =	2.025*e* − 1 (4.05*e* − 1) −	*5.475e − 1 (3.15e − 1)*
	10	3.451*e* − 1 (5.42*e* − 1) −	3.202*e* − 1 (3.44*e* − 1) −	3.441*e* − 1 (2.73*e* − 1) −	4.489*e* − 1 (1.46*e* − 2) =	3.126*e* − 1 (6.77*e* − 1) −	2.644*e* − 1 (4.07*e* − 1) −	*4.912e − 1 (4.38e − 1)*
	15	1.974*e* − 1 (3.95*e* − 1) −	3.644*e* − 1 (4.57*e* − 1) −	4.174*e* − 1 (3.61*e* − 1) −	4.465*e* − 1 (3.15*e* − 2) −	3.988*e* − 1 (5.66*e* − 1) =	8.995*e* − 2 (1.80*e* − 1) =	*9.879e − 1 (6.17e − 2)*

DTLZ4	3	5.523*e* − 1 (1.70*e* − 3) +	5.047*e* − 1 (9.30*e* − 2) +	5.262*e* − 1 (1.40*e* − 2) +	5.545*e* − 1 (1.16*e* − 3) +	*5.774e − 1 (5.25e − 4)* +	4.774*e* − 1 (2.97*e* − 4) −	4.954*e* − 1 (1.03*e* − 1)
	5	7.701*e* − 1 (8.65*e* − 3) −	7.411*e* − 1 (2.30*e* − 2) −	6.074*e* − 1 (3.15*e* − 2) −	7.697*e* − 1 (1.12*e* − 2) −	7.582*e* − 1 (7.73*e* − 3) =	*8.139e − 1 (1.11e − 3) =*	7.814*e* − 1 (8.95*e* − 3)
	8	9.073*e* − 1 (8.10*e* − 3) −	7.530*e* − 1 (1.22*e* − 1) −	7.371*e* − 1 (4.85*e* − 2) −	8.949*e* − 1 (1.04*e* − 2) −	7.861*e* − 1 (8.53*e* − 3) −	*9.352e − 1 (2.18e − 3) +*	9.113*e* − 1 (6.95*e* − 3)
	10	*9.419e − 1 (5.46e − 3)* =	7.469*e* − 1 (8.64*e* − 2) −	6.235*e* − 1 (8.62*e* − 2) −	9.273*e* − 1 (1.46*e* − 2) =	8.010*e* − 1 (9.28*e* − 3) −	8.615*e* − 1 (5.74*e* − 3) −	9.396*e* − 1 (6.00*e* − 3)
	15	9.857*e* − 1 (3.51*e* − 3) =	5.269*e* − 1 (2.15*e* − 1) −	5.219*e* − 1 (1.44*e* − 1) −	9.827*e* − 1 (3.77*e* − 3) =	7.948*e* − 1 (2.26*e* − 2) −	9.090*e* − 1 (1.72*e* − 3) −	*9.875e − 1 (8.11e − 4)*

DTLZ7	3	2.000*e* − 1 (1.86*e* − 2) −	2.431*e* − 1 (8.61*e* − 3) −	2.454*e* − 1 (1.07*e* − 2) −	2.025*e* − 1 (1.83*e* − 2) −	2.696*e* − 1 (2.61*e* − 3) −	2.029*e* − 1 (4.40*e* − 4) =	*2.526e − 1 (6.40e − 3)*
	5	8.128*e* − 2 (2.62*e* − 2) −	6.448*e* − 2 (1.72*e* − 2) −	5.921*e* − 2 (2.54*e* − 2) −	1.381*e* − 1 (1.73*e* − 2) =	*2.464e − 1 (1.26e − 2)* +	2.024*e* − 1 (7.68*e* − 3) +	1.320*e* − 1 (2.08*e* − 2)
	8	1.300*e* − 2 (1.32*e* − 2) −	2.252*e* − 3 (3.69*e* − 3) −	4.428*e* − 2 (3.69*e* − 2) −	2.967*e* − 2 (3.68*e* − 2) −	*1.320e − 1 (6.49e − 3)* +	5.777*e* − 2 (2.30*e* − 2) −	9.463*e* − 2 (1.67*e* − 2)
	10	7.479*e* − 4 (1.59*e* − 3) −	9.219*e* − 2 (9.50*e* − 3) =	5.648*e* − 2 (1.25*e* − 2) −	6.538*e* − 4 (3.97*e* − 4) −	6.737*e* − 2 (1.45*e* − 2) −	7.533*e* − 2 (7.97*e* − 3) =	*8.100e − 2 (1.87e − 3)*
	15	1.881*e* − 4 (4.00*e* − 4) −	4.649*e* − 2 (6.18*e* − 3) =	5.322*e* − 3 (4.12*e* − 5) −	5.973*e* − 7 (3.38*e* − 7) −	7.323*e* − 3 (1.10*e* − 5) −	4.484*e* − 2 (7.19*e* − 5) −	*5.400e − 2 (3.06e − 4)*

*w*/*l*/*t*		1/20/4	1/21/3	1/22/2	2/15/8	3/15/7	2/12/11	

**Table 10:** The statistical results (mean and standard deviation) of the HV values obtained by RVEA, NSGA-III, SPEAR, MOEA/DD, Two_Arch2, MOEA/DVA, and OBEA on WFG test suits. The best results are italicized.

Problem	M	RVEA	NSGA-III	SPEAR	MOEA/DD	Two_Arch2	MOEA/DVA	OBEA
WFG1	3	3.633*e* − 1 (1.84*e* − 2) −	3.558*e* − 1 (1.13*e* − 2) −	9.425*e* − 2 (1.90*e* − 2) −	2.823*e* − 1 (2.02*e* − 2) −	4.214*e* − 1 (1.71*e* − 2) −	3.291*e* − 1 (9.59*e* − 3) −	*7.613e − 1 (3.46e − 2)*
	5	3.118*e* − 1 (1.09*e* − 2) −	3.074*e* − 1 (3.49*e* − 3) −	2.180*e* − 1 (1.37*e* − 2) −	2.866*e* − 1 (5.40*e* − 3) −	3.935*e* − 1 (2.47*e* − 2) −	4.054*e* − 1 (1.28*e* − 2) −	*7.811e − 1 (6.25e − 1)*
	8	2.606*e* − 1 (1.58*e* − 2) =	2.596*e* − 1 (6.14*e* − 3) −	2.390*e* − 1 (5.72*e* − 3) −	2.433*e* − 1 (7.23*e* − 3) −	2.833*e* − 1 (1.30*e* − 2) −	3.131*e* − 1 (1.20*e* − 2) −	*4.913e − 1 (1.18e − 1)*
	10	2.351*e* − 1 (1.24*e* − 2) −	2.335*e* − 1 (5.63*e* − 3) −	2.219*e* − 1 (1.25*e* − 3) −	2.206*e* − 1 (1.59*e* − 3) −	2.649*e* − 1 (5.49*e* − 3) −	2.597*e* − 1 (1.16*e* − 2) −	*5.612e − 1 (4.13e − 2)*
	15	2.594*e* − 1 (4.63*e* − 2) −	2.615*e* − 1 (3.43*e* − 2) −	2.970*e* − 1 (1.67*e* − 2) −	1.884*e* − 1 (3.51*e* − 3) −	2.198*e* − 1 (9.17*e* − 3) −	2.240*e* − 1 (6.26*e* − 3) −	*9.913e − 1 (3.14e − 4)*

WFG2	3	8.442*e* − 1 (1.27*e* − 2) −	8.774*e* − 1 (1.01*e* − 2) −	7.574*e* − 1 (1.86*e* − 2) −	8.912*e* − 1 (1.43*e* − 2) =	*9.075e − 1 (5.74e − 3) =*	8.170*e* − 1 (4.90*e* − 3) −	8.837*e* − 1 (6.87*e* − 3)
	5	8.536*e* − 1 (1.88*e* − 2) −	8.712*e* − 1 (1.00*e* − 2) −	7.385*e* − 1 (1.59*e* − 2) −	8.983*e* − 1 (1.79*e* − 2) =	*9.584e − 1 (5.75e − 3) +*	9.556*e* − 1 (5.00*e* − 3) +	8.870*e* − 1 (1.17*e* − 2)
	8	8.454*e* − 1 (3.38*e* − 2) −	8.839*e* − 1 (1.82*e* − 2) −	8.297*e* − 1 (2.12*e* − 2) −	8.911*e* − 1 (2.59*e* − 2) −	*9.564e − 1 (2.12e − 3) =*	9.501*e* − 1 (1.86*e* − 2) =	*9.106e − 1 (1.58e − 2)*
	10	8.357*e* − 1 (2.69*e* − 2) −	8.666*e* − 1 (1.34*e* − 2) −	7.782*e* − 1 (2.05*e* − 2) −	8.544*e* − 1 (2.99*e* − 2) −	9.034*e* − 1 (7.76*e* − 3) =	9.083*e* − 1 (1.15*e* − 2) =	*9.086e − 1 (1.56e − 2)*
	15	8.429*e* − 1 (5.49*e* − 2) −	9.237*e* − 1 (2.74*e* − 2) −	9.075*e* − 1 (2.17*e* − 2) −	8.739*e* − 1 (3.97*e* − 2) −	9.390*e* − 1 (9.77*e* − 3) −	9.503*e* − 1 (4.19*e* − 3) =	9.543*e* − 1 (1.07*e* − 2)

WFG3	3	2.882*e* − 1 (1.28*e* − 2) −	3.246*e* − 1 (7.96*e* − 3) −	2.426*e* − 1 (2.19*e* − 2) −	3.246*e* − 1 (3.25*e* − 3) =	3.690*e* − 1 (5.93*e* − 3) =	*3.813e − 1 (3.53e − 3) =*	3.475*e* − 1 (8.44*e* − 3)
	5	9.344*e* − 3 (1.16*e* − 2) −	1.625*e* − 2 (1.19*e* − 2) −	2.808*e* − 3 (6.30*e* − 3) −	*4.313e − 1 (1.52e − 3) +*	1.522*e* − 1 (1.06*e* − 2) −	3.488*e* − 2 (9.22*e* − 3) −	1.769*e* − 1(1.28*e* − 3)
	8	1.301*e* − 1 (3.61*e* − 2) =	*1.372e − 1 (6.35e − 2) =*	1.126*e* − 1 (2.45*e* − 3) =	1.087*e* − 1 (3.20*e* − 2) =	1.124*e* − 1 (5.62*e* − 3) =	1.012*e* − 1 (5.32*e* − 3) =	1.131*e* − 1 (2.43*e* − 3)
	10	5.172*e* − 1 (4.77*e* − 2) −	5.768*e* − 1 (5.32*e* − 2) −	5.616*e* − 1 (2.77*e* − 2) =	5.090*e* − 1 (2.43*e* − 2) −	4.361*e* − 1 (6.33*e* − 2) −	5.513*e* − 1 (3.35*e* − 2) −	*6.945e − 1 (4.55e − 2)*
	15	2.757*e* − 1 (3.12*e* − 2) =	2.313*e* − 1 (4.66*e* − 2) =	3.331*e* − 1 (4.66*e* − 2) =	3.152*e* − 1 (8.52*e* − 2) =	3.033*e* − 1 (2.69*e* − 2) =	3.133*e* − 1 (2.66*e* − 2) =	*3.455e − 1 (4.69e − 2)*

WFG4	3	4.907*e* − 1 (4.55*e* − 3) −	5.035*e* − 1 (3.55*e* − 3) −	4.660*e* − 1 (6.87*e* − 3) −	5.015*e* − 1 (3.38*e* − 3) −	4.472*e* − 1 (2.18*e* − 3) −	*5.523e − 1 (1.71e − 3) =*	5.107*e* − 1 (2.91*e* − 3)
	5	6.544*e* − 1 (8.62*e* − 3) −	6.438*e* − 1 (8.01*e* − 3) −	6.362*e* − 1 (8.51*e* − 3) −	6.722*e* − 1 (1.28*e* − 2) −	*7.025e − 1 (5.47e − 3) =*	6.561*e* − 1 (1.07*e* − 2) −	6.785*e* − 1 (5.15*e* − 3)
	8	7.043*e* − 1 (2.18*e* − 2) −	7.360*e* − 1 (1.47*e* − 2) −	7.165*e* − 1 (1.38*e* − 2) −	6.954*e* − 1 (2.47*e* − 2) −	6.833*e* − 1 (7.37*e* − 3) −	6.933*e* − 1 (1.57*e* − 2) −	*7.707e − 1 (7.74e − 3)*
	10	7.294*e* − 1 (2.64*e* − 2) −	7.236*e* − 1 (1.56*e* − 2) −	7.459*e* − 1 (1.43*e* − 2) −	7.074*e* − 1 (2.30*e* − 2) =	6.634*e* − 1 (1.50*e* − 2) −	7.249*e* − 1 (2.00*e* − 2) =	*7.690e − 1 (1.19e − 2)*
	15	7.800*e* − 1 (3.65*e* − 2) −	6.974*e* − 1 (3.34*e* − 2) −	7.657*e* − 1 (3.12*e* − 2) −	7.673*e* − 1 (2.42*e* − 2) −	6.192*e* − 1 (6.80*e* − 3) −	7.177*e* − 1 (1.13*e* − 2) −	8.486*e* − 1 (1.13*e* − 2)

WFG5	3	4.720*e* − 1 (5.52*e* − 3) −	4.888*e* − 1 (5.08*e* − 3) −	4.598*e* − 1 (7.03*e* − 3) −	4.955*e* − 1 (4.10*e* − 3) =	5.191*e* − 1 (2.57*e* − 3) =	*5.314e − 1 (3.57e − 3) =*	4.939*e* − 1 (4.50*e* − 3)
	5	5.954*e* − 1 (9.61*e* − 3) −	5.787*e* − 1 (9.35*e* − 3) −	*6.466e − 1 (9.90e − 3) +*	6.395*e* − 1 (7.09*e* − 3) =	6.730*e* − 1 (5.44*e* − 3) =	5.462*e* − 1 (1.73*e* − 2) −	6.303*e* − 1 (9.01*e* − 3)
	8	6.320*e* − 1 (2.19*e* − 2) −	6.478*e* − 1 (1.57*e* − 2) −	6.621*e* − 1 (1.52*e* − 2) −	6.307*e* − 1 (2.10*e* − 2) −	6.317*e* − 1 (9.55*e* − 3) −	7.035*e* − 1 (1.41*e* − 2) =	*7.098e − 1 (1.44e − 2)*
	10	5.892*e* − 1 (2.82*e* − 2) −	6.020*e* − 1 (1.85*e* − 2) −	*6.577e − 1 (9.98e − 3) +*	5.839*e* − 1 (2.39*e* − 2) −	5.674*e* − 1 (1.14*e* − 2) −	5.944*e* − 1 (6.85*e* − 3) −	6.377*e* − 1 (1.79*e* − 2)
	15	6.375*e* − 1 (4.07*e* − 2) −	6.663*e* − 1 (4.52*e* − 2) −	6.915*e* − 1 (1.34*e* − 2) −	6.406*e* − 1 (5.40*e* − 2) −	4.687*e* − 1 (2.33*e* − 2) −	6.781*e* − 1 (5.97*e* − 3) −	*7.498e − 1 (2.03e − 2)*

WFG6	3	4.414*e* − 1 (8.75*e* − 3) −	4.665*e* − 1 (1.07*e* − 2) −	4.412*e* − 1 (2.98*e* − 2) −	4.390*e* − 1 (3.29*e* − 2) −	5.040*e* − 1 (6.53*e* − 3) =	5.012*e* − 1 (5.62*e* − 4) −	*5.160e − 1 (1.14e − 2)*
	5	5.611*e* − 1 (1.53*e* − 2) −	5.403*e* − 1 (1.56*e* − 2) −	5.370*e* − 1 (5.69*e* − 2) −	5.935*e* − 1 (1.83*e* − 2) −	6.488*e* − 1 (8.33*e* − 3) −	5.667*e* − 1 (1.95*e* − 2) −	*6.927e − 1 (1.50e − 2)*
	8	5.571*e* − 1 (4.80*e* − 2) −	6.108*e* − 1 (2.64*e* − 2) −	6.491*e* − 1 (7.53*e* − 2) =	6.072*e* − 1 (2.98*e* − 2) −	5.989*e* − 1 (1.95*e* − 2) −	5.970*e* − 1 (2.33*e* − 2) −	*7.598e − 1 (1.81e − 2)*
	10	5.560*e* − 1 (2.31*e* − 2) −	5.458*e* − 1 (2.06*e* − 2) −	5.821*e* − 1 (2.81*e* − 2) −	5.751*e* − 1 (2.66*e* − 2) −	5.315*e* − 1 (1.12*e* − 2) −	5.909*e* − 1 (2.63*e* − 2) −	*7.557e − 1 (2.25e − 2)*
	15	6.181*e* − 1 (4.76*e* − 2) −	6.155*e* − 1 (5.22*e* − 2) −	6.656*e* − 1 (2.79*e* − 2) −	6.888*e* − 1 (2.87*e* − 2) −	4.290*e* − 1 (1.47*e* − 2) −	5.285*e* − 1 (5.15*e* − 2) −	*7.229e − 1 (3.09e − 2)*

WFG7	3	4.701*e* − 1 (1.52*e* − 2) −	5.005*e* − 1 (6.55*e* − 3) −	4.734*e* − 1 (9.21*e* − 3) −	5.111*e* − 1 (1.29*e* − 2) −	5.601*e* − 1 (1.03*e* − 3) +	*5.648e − 1 (9.74e − 4) +*	5.148*e* − 1 (2.33*e* − 3)
	5	6.106*e* − 1 (1.89*e* − 2) −	5.821*e* − 1 (1.50*e* − 2) −	5.966*e* − 1 (1.46*e* − 2) −	6.506*e* − 1 (1.39*e* − 2) −	*7.271e − 1 (2.98e − 3) =*	6.129*e* − 1 (2.46*e* − 2) −	7.182*e* − 1 (5.40*e* − 3)
	8	6.679*e* − 1 (3.37*e* − 2) −	6.735*e* − 1 (2.44*e* − 2) −	6.340*e* − 1 (1.67*e* − 2) −	6.250*e* − 1 (2.70*e* − 2) −	6.752*e* − 1 (1.15*e* − 2) −	6.735*e* − 1 (2.38*e* − 3) −	*8.113e − 1 (6.13e − 3)*
	10	6.658*e* − 1 (2.40*e* − 2) −	6.636*e* − 1 (2.32*e* − 2) −	6.574*e* − 1 (1.76*e* − 2) −	6.087*e* − 1 (2.39*e* − 2) −	6.128*e* − 1 (1.43*e* − 2) −	6.799*e* − 1 (4.66*e* − 3) −	*8.130e − 1 (4.13e − 2)*
	15	6.960*e* − 1 (5.44*e* − 2) −	6.274*e* − 1 (5.04*e* − 2) −	7.397*e* − 1 (2.14*e* − 2) −	6.825*e* − 1 (3.93*e* − 2) −	5.063*e* − 1 (2.81*e* − 2) −	6.691*e* − 1 (2.88*e* − 2) −	*8.167e − 1 (4.01e − 2)*

WFG8	3	4.329*e* − 1 (6.27*e* − 3) −	4.533*e* − 1 (5.50*e* − 3) −	3.919*e* − 1 (9.86*e* − 3) −	4.260*e* − 1 (1.83*e* − 2) −	4.883*e* − 1 (3.72*e* − 3) −	4.968*e* − 1 (3.89*e* − 3) −	*5.122e − 1 (5.15e − 2)*
	5	5.465*e* − 1 (9.83*e* − 3) −	5.398*e* − 1 (1.22*e* − 2) −	5.025*e* − 1 (1.11*e* − 2) −	5.238*e* − 1 (2.02*e* − 2) −	6.189*e* − 1 (5.45*e* − 3) −	5.277*e* − 1 (1.40*e* − 2) −	*6.915e − 1 (3.63e − 3)*
	8	5.081*e* − 1 (7.05*e* − 2) −	6.039*e* − 1 (3.24*e* − 2) −	5.586*e* − 1 (4.46*e* − 2) −	5.236*e* − 1 (3.00*e* − 2) −	5.257*e* − 1 (6.61*e* − 3) −	4.231*e* − 1 (4.73*e* − 2) −	*6.124e − 1 (6.71e − 2)*
	10	5.321*e* − 1 (6.01*e* − 2) −	5.568*e* − 1 (2.18*e* − 2) −	6.051*e* − 1 (1.51*e* − 2) =	5.069*e* − 1 (2.76*e* − 2) −	4.585*e* − 1 (2.08*e* − 2) −	4.168*e* − 1 (2.98*e* − 2) −	*7.184e − 1 (5.17e − 2)*
	15	5.909*e* − 1 (1.26*e* − 1) =	4.682*e* − 1 (8.94*e* − 2) −	6.588*e* − 1 (2.44*e* − 2) −	6.781*e* − 1 (6.17*e* − 2) −	3.421*e* − 1 (1.90*e* − 2) −	3.675*e* − 1 (9.47*e* − 2) −	*7.841e − 1 (5.17e − 2)*

WFG9	3	4.234*e* − 1 (1.68*e* − 2) −	4.534*e* − 1 (1.39*e* − 2) −	4.474*e* − 1 (6.05*e* − 3) −	4.873*e* − 1 (1.62*e* − 2) −	5.234*e* − 1 (2.08*e* − 2) −	4.940*e* − 1 (2.47*e* − 3) −	*5.611e − 1 (6.12e − 3)*
	5	5.346*e* − 1 (2.17*e* − 2) −	5.383*e* − 1 (2.10*e* − 2) −	6.423*e* − 1 (6.23*e* − 3) =	5.869*e* − 1 (2.65*e* − 2) −	*6.733e − 1 (1.54e − 2) =*	6.324*e* − 1 (1.38*e* − 2) =	6.655*e* − 1 (4.17*e* − 2)
	8	5.663*e* − 1 (4.39*e* − 2) −	5.807*e* − 1 (4.29*e* − 2) −	6.484*e* − 1 (1.12*e* − 2) =	5.386*e* − 1 (3.35*e* − 2) −	6.204*e* − 1 (2.10*e* − 2) −	6.512*e* − 1 (1.32*e* − 2) =	*7.012e − 1 (6.32e − 2)*
	10	5.373*e* − 1 (3.41*e* − 2) −	5.656*e* − 1 (3.86*e* − 2) −	6.499*e* − 1 (1.96*e* − 2) =	5.248*e* − 1 (4.63*e* − 2) −	5.793*e* − 1 (3.06*e* − 2) −	6.617*e* − 1 (1.86*e* − 2) =	*6.791e − 1 (4.17e − 2)*
	15	5.387*e* − 1 (7.57*e* − 2) −	6.343*e* − 1 (5.56*e* − 2) −	6.232*e* − 1 (3.56*e* − 2) −	5.315*e* − 1 (6.46*e* − 2) −	4.703*e* − 1 (1.29*e* − 2) −	5.670*e* − 1 (5.39*e* − 3) −	*7.824e − 1 (3.42e − 2)*

*w*/*l*/*t*		0/41/4	0/43/2	2/35/8	1/36/8	2/31/12	2/30/13	

**Table 11:** Performance comparison of OBEA and its two variants on DTLZ and WFG test suites in terms of HV.

Problem	M	OBEA-lin	OBEA-exp	OBEA-sig
DTLZ1	3	7.2247*e* − 1 (1.39*e* − 1) −	6.2997*e* − 1 (2.95*e* − 1) −	*8.471e − 1 (6.13e − 3)*
	5	6.7003*e* − 1 (3.78*e* − 1) −	5.7003*e* − 1 (3.78*e* − 1) −	*9.763e − 1 (4.15e − 4)*
	8	2.7315*e* − 1 (5.46*e* − 2) −	2.1610*e* − 1 (3.96*e* − 1) −	*9.524e − 1 (6.84e − 3)*
	10	7.5060*e* − 1 (1.06*e* − 1) −	6.5552*e* − 1 (3.17*e* − 1) −	*9.576e − 1 (4.18e − 3)*
	15	2.3922*e* − 1 (2.08*e* − 1) −	1.6218*e* − 1 (2.24*e* − 1) −	*9.456e − 1 (4.46e − 6)*

DTLZ2	3	1.1140*e* − 1 (2.84*e* − 2) −	4.7777*e* − 1 (3.06*e* − 2) −	*5.563e − 1 (5.70e − 4)*
	5	1.5081*e* − 1 (3.88*e* − 2) −	6.1838*e* − 1 (6.07*e* − 2) −	*7.829e − 1 (3.48e − 3)*
	8	3.2693*e* − 1 (1.91*e* − 2) −	8.0224*e* − 1 (2.08*e* − 2) =	*8.981e − 1 (4.96e − 3)*
	10	1.9501*e* − 1 (4.99*e* − 2) −	5.0579*e* − 1 (8.63*e* − 2) −	*9.071e − 1 (1.22e − 2)*
	15	1.7267*e* − 1 (4.04*e* − 2) −	1.1953*e* − 1 (3.14*e* − 2) −	*9.459e − 1 (7.02e − 3)*

DTLZ3	3	1.7451*e* − 3 (9.56*e* − 3) −	1.5717*e* − 1 (3.36*e* − 4) −	*5.212e − 1 (4.53e − 2)*
	5	4.5189*e* − 1 (1.68*e* − 1) −	3.6816*e* − 1 (1.38*e* − 3) −	*7.688e − 1 (5.15e − 2)*
	8	*7.0697e − 1 (6.81e − 2) +*	4.4701*e* − 1 (2.07*e* − 2) −	5.475*e* − 1 (3.15*e* − 1)
	10	6.5542*e* − 1 (5.27*e* − 2) +	*7.3079e − 1 (8.45e − 3) +*	4.912*e* − 1 (4.38*e* − 1)
	15	2.5727*e* − 1 (1.01*e* − 2) −	5.5234*e* − 1 (5.97*e* − 2) −	*9.879e − 1 (6.17e − 2)*

DTLZ4	3	1.6104*e* − 1 (3.76*e* − 2) −	4.6278*e* − 1 (1.06*e* − 1) −	*4.954e − 1 (1.03e − 1)*
	5	2.0649*e* − 1 (4.92*e* − 2) −	6.4816*e* − 1 (5.07*e* − 2) −	*7.814e − 1 (8.95e − 3)*
	8	3.4719*e* − 1 (6.19*e* − 3) −	8.2327*e* − 1 (4.48*e* − 2) =	*9.113e − 1 (6.95e − 3)*
	10	1.9669*e* − 1 (6.25*e* − 2) −	6.7233*e* − 1 (6.52*e* − 2) −	*9.396e − 1 (6.00e − 3)*
	15	1.7155*e* − 1 (4.37*e* − 2) −	1.1721*e* − 1 (9.91*e* − 2) −	*9.875e − 1 (8.11e − 4)*

DTLZ7	3	*2.5727e − 1 (1.01e − 2) =*	1.8185*e* − 1 (3.85*e* − 2) −	2.526*e* − 1 (6.40*e* − 3)
	5	*1.8367e − 1 (4.58e − 2) +*	1.1242*e* − 1 (5.40*e* − 2) −	1.320*e* − 1 (2.08*e* − 2)
	8	1.2285*e* − 2 (2.86*e* − 2) −	1.5925*e* − 2 (1.36*e* − 2) −	*9.463e − 2 (1.67e − 2)*
	10	1.2317*e* − 2(2.21*e* − 4) −	3.9912*e* − 2 (8.81*e* − 3) −	*8.100e − 2 (1.87e − 3)*
	15	1.0056*e* − 7 (1.82*e* − 7) −	1.3300*e* − 1 (4.90*e* − 2) −	*5.400e − 2 (3.06e − 4)*

WFG1	3	5.0301*e* − 1 (4.50*e* − 2) −	2.4845*e* − 1 (3.59*e* − 2) −	*7.613e − 1 (3.46e − 2)*
	5	3.9091*e* − 1 (4.40*e* − 2) −	2.8672*e* − 1 (3.36*e* − 2) −	*7.811e − 1 (6.25e − 1)*
	8	8.5770*e* − 1 (6.00*e* − 3) +	2.4956*e* − 1 (1.26*e* − 3) −	4.913*e* − 1 (1.18*e* − 1)
	10	3.0816*e* − 1 (3.93*e* − 2) −	2.3200*e* − 1 (2.02*e* − 2) −	*5.612e − 1 (4.13e − 2)*
	15	9.0611*e* − 1 (2.30*e* − 4) =	8.6141*e* − 2 (5.89*e* − 2) −	*9.913e − 1 (3.14e − 4)*

WFG2	3	*9.0441e − 1 (9.92e − 3) =*	8.3414*e* − 1 (5.40*e* − 2) −	8.837*e* − 1 (6.87*e* − 3)
	5	*9.5718e − 1 (2.01e − 2) =*	8.9478*e* − 1 (3.42*e* − 2) =	8.870*e* − 1 (1.17*e* − 2)
	8	8.9191*e* − 1 (2.13*e* − 2) =	8.9445*e* − 1 (3.75*e* − 2) =	*9.106e − 1 (1.58e − 2)*
	10	*9.6176e − 1 (6.39e − 2) =*	8.9860*e* − 1 (8.22*e* − 2) =	9.086*e* − 1 (1.56*e* − 2)
	15	9.2253*e* − 1 (6.05*e* − 2) =	4.3771*e* − 1 (1.93*e* − 1) −	*9.543e − 1 (1.07e − 2)*

WFG3	3	*3.7932e − 1 (9.83e − 3) =*	3.4173*e* − 1 (2.57*e* − 2) =	3.475*e* − 1 (8.44*e* − 3)
	5	*5.2190e − 1 (3.31e − 2) +*	3.4134*e* − 2 (3.78*e* − 2) −	1.769*e* − 1 (1.28*e* − 3)
	8	1.0221*e* − 1 (3.56*e* − 3) =	*1.4421e − 1 (3.31e − 3) =*	1.131*e* − 1 (2.43*e* − 3)
	10	5.3674*e* − 1 (5.52*e* − 2) =	5.3927*e* − 1 (3.50*e* − 3) =	*6.945e − 1 (4.55e − 2)*
	15	3.2132*e* − 1 (1.43*e* − 1) =	3.2479*e* − 1 (2.74*e* − 2) =	*3.455e − 1 (4.69e − 2)*

*w*/*l*/*t*		5/29/16	1/30/9	

**Table 12:** The statistical results of the HV obtained by OBEA, OBEA-APD, and OBEA-PBI. The best results are italicized.

Problem	M	DTLZ1	DTLZ2	WFG2	WFG4
OBEA	3	*8.471e − 1 (6.13e − 3)*	*5.563e − 1 (5.70e − 4)*	8.837*e* − 1 (6.87*e* − 3)	*5.107e − 1 (2.91e − 3)*
	5	*9.761e − 1 (4.15e − 4)*	*7.829e − 1 (3.48e − 3)*	8.870*e* − 1 (1.17*e* − 2)	6.785*e* − 1 (5.15*e* − 3)
	8	*9.524e − 1 (6.84e − 3)*	*8.981e − 1 (4.96e − 3)*	*9.106e − 1 (1.58e − 2)*	*7.707e − 1 (7.74e − 3)*
	10	*9.571e − 1 (4.18e − 3)*	*9.071e − 1 (1.22e − 2)*	9.086*e* − 1 (1.56*e* − 2)	*7.690e − 1 (1.19e − 2)*
	15	9.456*e* − 1 (4.46*e* − 6)	9.459*e* − 1 (7.02*e* − 3)	*9.543e − 1 (1.07e − 2)*	*8.486e − 1 (1.13e − 2)*

OBEA-APD	3	6.299*e* − 1 (2.95*e* − 1) −	4.777*e* − 1 (3.06*e* − 2) −	8.341*e* − 1 (5.40*e* − 2) −	5.353*e* − 1 (2.85*e* − 3) −
	5	5.700*e* − 1 (3.78*e* − 1) −	6.183*e* − 1 (6.07*e* − 2) −	*9.044e − 1 (9.92e − 3) =*	*7.116e − 1 (7.46e − 3) =*
	8	9.161*e* − 1 (3.96*e* − 1) =	8.022*e* − 1 (2.08*e* − 2) −	8.571*e* − 1 (2.01*e* − 2) −	7.021*e* − 1 (2.74*e* − 2) =
	10	6.555*e* − 1 (3.17*e* − 1) −	9.057*e* − 1 (8.63*e* − 2) =	8.986*e* − 1 (8.22*e* − 2) =	6.675*e* − 1 (8.16*e* − 2) −
	15	*9.521e − 1 (2.24e − 1) =*	9.195*e* − 1 (3.14*e* − 2) −	4.377*e* − 1 (1.93*e* − 1) −	5.933*e* − 1 (1.27*e* − 2) −

OBEA-PBI	3	7.224*e* − 1 (1.39*e* − 1) −	4.114*e* − 1 (2.84*e* − 2) −	8.047*e* − 1 (3.42*e* − 2) −	4.479*e* − 1 (2.57*e* − 2) −
	5	6.700*e* − 1 (3.78*e* − 1) −	7.508*e* − 1 (3.88*e* − 2) −	8.014*e* − 1 (3.75*e* − 2) −	5.121*e* − 1 (4.26*e* − 2) −
	8	9.731*e* − 1 (5.46*e* − 2) =	7.269*e* − 1 (1.91*e* − 2) −	8.919*e* − 1 (2.13*e* − 2) =	6.299*e* − 1 (4.47*e* − 2) −
	10	7.506*e* − 1 (1.06*e* − 1) −	9.050*e* − 1 (4.99*e* − 2) =	*9.176e − 1 (6.39e − 2)* =	4.798*e* − 1 (5.73*e* − 2) −
	15	9.392*e* − 1 (2.08*e* − 1) =	9.026*e* − 1 (4.04*e* − 2) −	9.225*e* − 1 (6.05*e* − 2) =	6.059*e* − 1 (1.90*e* − 2) −

**Table 13:** The statistical results of the HV obtained by various OBEAs. The best results are italicized.

Problem	M	OBEA1	OBEA2	OBEA3	OBEA
DTLZ1	3	6.1214*e* − 1 (3.24*e* − 2) −	1.0640*e* − 1 (5.12*e* − 2) −	6.0619*e* − 1 (4.74*e* − 2) −	*8.471e − 1 (6.13e − 3)*
	5	5.9038*e* − 1 (2.21*e* − 2) −	6.8560*e* − 1 (7.88*e* − 3) −	7.4080*e* − 1 (5.74*e* − 4) −	*9.763e − 1 (4.15e − 4)*
	8	5.9182*e* − 1 (4.12*e* − 2) −	5.2621*e* − 1 (4.82*e* − 2) −	4.7633*e* − 1 (6.07*e* − 4) −	*9.524e − 1 (6.84e − 3)*
	10	8.9323*e* − 1 (2.66*e* − 2) −	6.9613*e* − 1 (3.94*e* − 1) −	7.8192*e* − 1 (6.59*e* − 4) −	*9.576e − 1 (4.18e − 3)*
	15	7.8893*e* − 1 (5.45*e* − 3) −	8.5401*e* − 1 (6.84*e* − 1) =	6.8614*e* − 1 (5.54*e* − 3) −	*9.456e − 1 (4.46e − 6)*

DTLZ2	3	*5.7673e − 1 (2.42e − 4) =*	5.6556*e* − 1 (9.98*e* − 4) =	5.1138*e* − 1 (7.82*e* − 4) −	5.563*e* − 1 (5.70*e* − 4)
	5	7.7404*e* − 1 (3.05*e* − 4) −	6.3986*e* − 1 (4.48*e* − 3) −	7.6299*e* − 1 (6.20*e* − 3) −	*7.829e − 1 (3.48e − 3)*
	8	8.8491*e* − 1 (6.94*e* − 3) =	7.1621*e* − 1 (4.14*e* − 2) −	6.2045*e* − 1 (3.92*e* − 3) −	*8.981e − 1 (4.96e − 3)*
	10	*9.3184e − 1 (3.75e − 3) =*	8.7840*e* − 1 (7.51*e* − 2) −	7.1978*e* − 1 (1.62*e* − 3) −	9.071*e* − 1 (1.22*e* − 2)
	15	8.2096*e* − 1 (2.89*e* − 2) −	8.2838*e* − 1 (7.58*e* − 2) −	6.7601*e* − 1 (3.08*e* − 3) −	*9.459e − 1 (7.02e − 3)*

DTLZ3	3	*5.4667e − 1 (4.62e − 1) =*	5.0214*e* − 1 (6.47*e* − 1) =	2.0327*e* − 1 (6.63*e* − 1) −	5.212*e* − 1 (4.53*e* − 2)
	5	6.2447*e* − 1 (2.25*e* − 1) −	6.7172*e* − 1 (4.55*e* − 1) −	4.4995*e* − 1 (2.67*e* − 1) −	*7.688e − 1 (5.15e − 2)*
	8	4.6312*e* − 1 (3.12*e* − 2) =	5.7638*e* − 1 (3.66*e* − 2) =	2.0254*e* − 1 (4.05*e* − 1) −	5.475*e* − 1 (3.15*e* − 1)
	10	3.4412*e* − 1 (2.73*e* − 1) −	4.5211*e* − 1 (6.42*e* − 1) −	2.6443*e* − 1 (4.07*e* − 1) −	*4.912e − 1 (4.38e − 1)*
	15	8.1743*e* − 1 (3.61*e* − 1) =	6.7614*e* − 1 (5.42*e* − 1) −	6.9950*e* − 2 (1.80*e* − 1) −	*9.879e − 1 (6.17e − 2)*

WFG1	3	6.1470*e* − 1 (6.81*e* − 2) −	*5.0581e − 1 (4.05e − 2) −*	3.6909*e* − 1 (5.93*e* − 3) −	*7.613e − 1 (3.46e − 2)*
	5	5.0201*e* − 1 (2.28*e* − 2) −	4.3295*e* − 1 (2.37*e* − 2) −	1.5220*e* − 1 (1.06*e* − 2) −	*7.811e − 1 (6.25e − 1)*
	8	4.1424*e* − 1 (2.95*e* − 2) =	2.8761*e* − 1 (1.70*e* − 2) −	1.1243*e* − 1 (5.62*e* − 3) −	*4.913e − 1 (1.18e − 1)*
	10	4.6201*e* − 1 (2.57*e* − 2) =	2.7123*e* − 1 (5.51*e* − 2) −	4.3613*e* − 1 (6.33*e* − 2) =	*5.612e − 1 (4.13e − 2)*
	15	8.2429*e* − 1 (2.51*e* − 2) −	8.0893*e* − 1 (2.18*e* − 2) −	6.0337*e* − 1 (2.69*e* − 2) −	*9.913e − 1 (3.14e − 4)*

WFG2	3	8.0484*e* − 1 (3.64*e* − 3) −	8.0997*e* − 1 (2.71*e* − 3) =	5.4720*e* − 1 (2.18*e* − 3) −	*8.837e − 1 (6.87e − 3)*
	5	*9.0383e − 1 (3.83e − 3) =*	8.3424*e* − 1 (9.68*e* − 3) =	7.0257*e* − 1 (5.47*e* − 3) −	8.870*e* − 1 (1.17*e* − 2)
	8	8.3108*e* − 1 (6.92*e* − 3) =	*9.1790e − 1 (1.42e − 2) =*	6.8330*e* − 1 (7.37*e* − 3) −	9.106*e* − 1 (1.58*e* − 2)
	10	8.3799*e* − 1 (6.61*e* − 3) =	8.9274*e* − 1 (1.80*e* − 2) −	6.6341*e* − 1 (1.50*e* − 2) −	*9.086e − 1 (1.56e − 2)*
	15	9.2971*e* − 1 (1.54*e* − 2) =	8.5648*e* − 1 (4.82*e* − 2) =	6.1924*e* − 1 (6.80*e* − 3) −	*9.543e − 1 (1.07e − 2)*

*w*/*l*/*t*		0/13/12	0/17/8	0/24/1	

## Data Availability

All data included in this study are available from the corresponding author upon request.
